# Vascular wall microenvironment: exosomes secreted by adventitial fibroblasts induced vascular calcification

**DOI:** 10.1186/s12951-023-02000-3

**Published:** 2023-09-04

**Authors:** Ming-Hui Zheng, Su-Kang Shan, Xiao Lin, Feng Xu, Feng Wu, Bei Guo, Fu-Xing-zi Li, Zhi-Ang Zhou, Yi Wang, Li-Min Lei, Ke-Xin Tang, Jia-Yue Duan, Yun-Yun Wu, Ye-Chi Cao, Xiao-Bo Liao, Ling-Qing Yuan

**Affiliations:** 1 Department of Metabolism and Endocrinology, National Clinical Research Center for Metabolic Diseases, the Second Xiangya Hospital, Central South University, Changsha, 410000 China; 2 Department of Radiology, the Second Xiangya Hospital, Central South University, Changsha, 410000 China; 3 Department of Pathology, the Second Xiangya Hospital, Central South University, Changsha, 410000 China; 4 Department of Cardiovascular Surgery, the Second Xiangya Hospital, Central South University, Changsha, 410000 China

**Keywords:** Exosomes, Vascular calcification, Microenvironment, High phosphorus, Adventitia fibroblasts, Vascular smooth muscle cells, miR-21-5p

## Abstract

Vascular calcification often occurs in patients with chronic renal failure (CRF), which significantly increases the incidence of cardiovascular events in CRF patients. Our previous studies identified the crosstalk between the endothelial cells (ECs) and vascular smooth muscle cells (VSMCs), and the paracrine effect of VSMCs, which regulate the calcification of VSMCs. Herein, we aim to investigate the effects of exosomes secreted by high phosphorus (HPi) -induced adventitial fibroblasts (AFs) on the calcification of VSMCs and the underlying mechanism, which will further elucidate the important role of AFs in high phosphorus vascular wall microenvironment. The conditioned medium of HPi-induced AFs promotes the calcification of VSMCs, which is partially abrogated by GW4869, a blocker of exosomes biogenesis or release. Exosomes secreted by high phosphorus-induced AFs (AFs^HPi^-Exos) show similar effects on VSMCs. miR-21-5p is enriched in AFs^HPi^-Exos, and miR-21-5p enhances osteoblast-like differentiation of VSMCs by downregulating cysteine-rich motor neuron 1 (Crim1) expression. AFs^HPi^-Exos and exosomes secreted by AFs with overexpression of miR-21-5p (AFs^miR21M^-Exos) significantly accelerate vascular calcification in CRF mice. In general, AFs^HPi^-Exos promote the calcification of VSMCs and vascular calcification by delivering miR-21-5p to VSMCs and subsequently inhibiting the expression of Crim1. Combined with our previous studies, the present experiment supports the theory of vascular wall microenvironment.

**Graphical Abstract:**

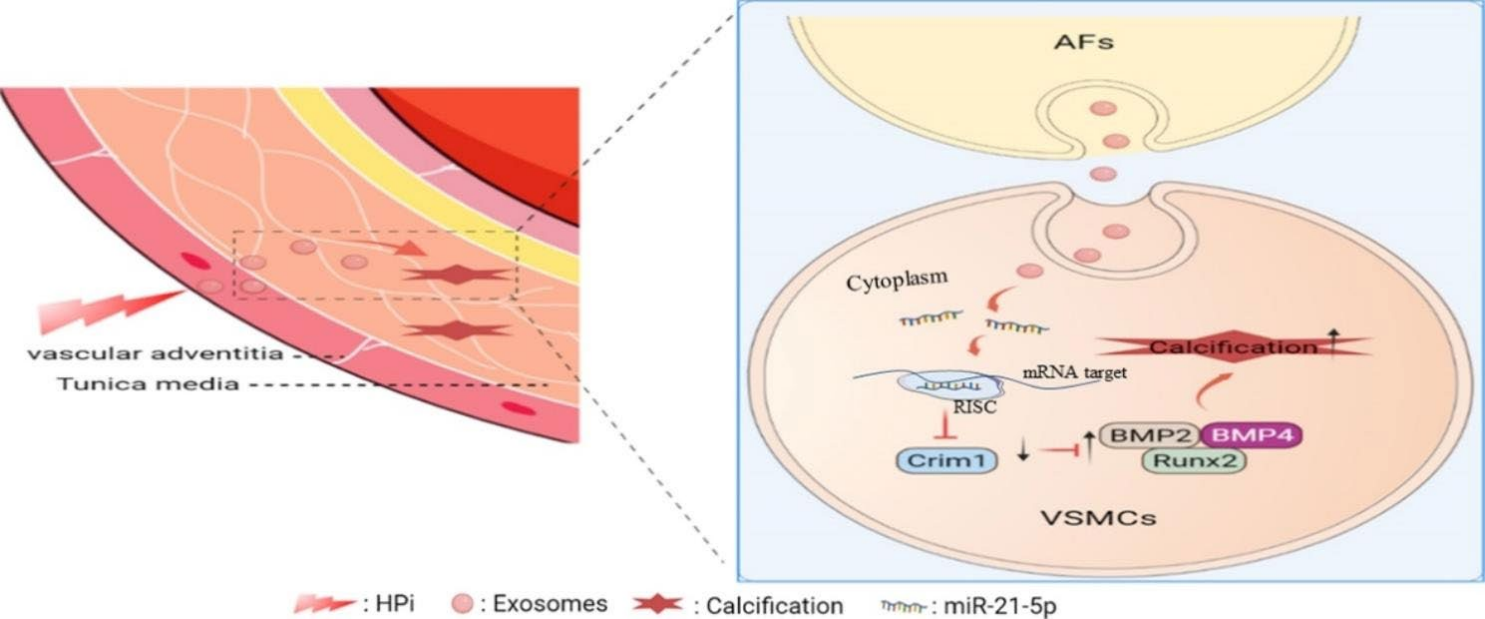

**Supplementary Information:**

The online version contains supplementary material available at 10.1186/s12951-023-02000-3.

## Introduction

Vascular calcification is a kind of ectopic mineralization, which occurs in the cardiovascular system and leads to decreased vascular wall elasticity and impaired vascular structural integrity. Vascular calcification often occurs in patients with chronic kidney disease, diabetes, atherosclerosis, hypertension, and other diseases, which significantly increases the risk of death from cardiovascular disease [1, 2]. Vascular calcification can occur in almost all types of arteries, such as the abdominal aorta, coronary artery, aortic valve, et al. [3–5]. Moreover, calcification occurs in both the intima and media of the artery wall. Intima calcification, also known as atherosclerotic calcification, is mainly manifested by inflammation and the deposition of lipids [6, 7]. Medial calcification refers to the trans-differentiation of vascular smooth muscle cells (VSMCs) into osteoblast-like cells under the stimulation of high phosphorus or high glucose [8–13]. However, the specific mechanism of the development of vascular calcification remains unclear.

Arterial calcification is often found in patients suffered from chronic kidney disease [14]. According to an epidemiological survey in 2017, the prevalence of chronic kidney disease is 9.1%. There are more than 600 million chronic kidney disease patients worldwide, and more than 1.2 million deaths are caused by chronic kidney disease every year [15]. Vascular calcification in patients with chronic renal failure (CRF) is mainly caused by metabolic disorders, such as hyperphosphatemia, hypercalcemia, and uremia [16]. Hyperphosphatemia in patients with advanced CRF is often based on impaired renal excretion of phosphate [17]. Therefore, high serum phosphate can be considered a vascular toxin [18, 19]. The deposition of calcium and phosphorus on the vascular wall can directly cause vascular calcification. In addition, phosphorus and calcium can individually or jointly promote the dysfunction and calcification of VSMCs [20]. High concentration of phosphorus can not only directly promote the phenotypic transformation of VSMCs into osteoblast-like cells, reduce the expression of smooth muscle cell markers and enhance the expression of osteochondrogenic genes [21], but also cause the calcification of VSMCs by increasing the apoptosis and mitochondrial oxidative stress of VSMCs, as well as the release of calcifying matrix vesicles [9, 22, 23]. Thi Nguyen et al. found that phosphate carrier (PiC) is important for mitochondrial phosphorus uptake and mediates superoxide production and calcification in VSMCs induced by high phosphorus [9]. Our recent study demonstrated that exosomes (Exos) secreted from ECs induced by high phosphate could significantly promote VSMCs calcification by enriching miR-670-3p in ECs-derived exosomes [24]. Li et al. reported that exosomal let-7b-5p derived from macrophage induced by high-phosphate is responsible for the vascular calcification in chronic kidney disease [25]. However, the pathogenesis of hyperphosphatemia induced vascular calcification in CRF patients is complex and unclear, so further studies are needed to elucidate the mechanism of its occurrence and development.

Vascular walls are composed of the intima, media, and adventitia, which are mainly composed of endothelial cells (ECs), VSMCs and adventitial fibroblasts (AFs) respectively [26]. Due to their adjacency in location, cells can communicate with each other in various physiological and pathological conditions. Recently, a large number of studies have focused on the communication between ECs and VSMCs. Our recent studies demonstrated the ECs original exosomes could mediate the intima and media communications [24, 27]. Moreover, exosomes secreted by VSMCs could regulate the calcification and ageing of adjacent VSMCs through a paracrine mechanism [2]. However, whether AFs could regulate the calcification is still unknown. If this phenomenon can be confirmed, the theory of vessel wall microenvironment will be constructed.

Cells can release various extracellular vesicles, which are mainly divided into exosomes, microvesicles, and apoptotic bodies according to their diameter and biological formation process [28]. Exosomes are extracellular vesicles originated from endosomal vesicles of the multivesicular bodies [29–31]. Exosomes, with a diameter of 30–150 nm, have a lipid bilayer structure and carry DNA, miRNA, mRNA, lipids, proteins, and other substances [32, 33]. The exosome membrane contains cholesterol, sphingolipid, ceramide, and other substances, which keep the structure of exosomes highly stable. After exosomes are released from cells, they enter the blood, urine, cerebrospinal fluid, saliva, breast milk, ascites, and other body fluids [34]. Exosomes are important media of intercellular communication and play an important role in the information transfer between cells [35]. In recent years, much literature has reported the important role of exosomes in the development of vascular calcification. Previous studies in our group have shown that exosomes secreted by melatonin-treated VSMCs transfer miR-204/miR-211 to adjacent VSMCs in a paracrine manner, alleviating vascular calcification and aging by regulating BMP2 [2]. In addition, plasma exosomes derived from end-stage renal disease patients could promote VSMCs calcification, while plasma exosomes from renal transplant recipients could partially attenuate VSMCs calcification [36]. A recent study by our group found that miR-670-3p is enriched in exosomes derived from ECs induced by high phosphorus (ECs^HPi^-Exos) and is essential for the ECs^HPi^-Exos-induced VSMCs calcification [24]. These studies indicated that exosomes play an important role in vascular calcification, which is of great interest to us to explore the role of AFs-derived exosomes in vascular calcification.

In our present study, we found that exosomes secreted by 3.5 mM inorganic phosphorus (HPi) induced AFs (AFs^HPi^-Exos) significantly promoted the calcification of VSMCs compared with exosomes secreted by 0.9 mM inorganic phosphorus (NPi) induced AFs (AFs^NPi^-Exos). After inhibiting the biogenesis or secretion of exosomes by AFs with GW4869, the pro-calcification effect was significantly weakened. The miR-21-5p level in AFs^HPi^-Exos was significantly upregulated compared with AFs^NPi^-Exos. Our results demonstrated that AFs^HPi^-Exos promoted the calcification of VSMCs by transferring miR-21-5p into VSMCs and inhibiting the expression of cysteine-rich motor neuron 1 (Crim1) in VSMCs. In conclusion, our study suggested that the high level of miR-21-5p carried by AFs^HPi^-Exos and its inhibition of the target gene Crim1 played an important role in the pathogenesis of vascular calcification in patients with CRF.

## Materials and methods

### Ethics statement

All experiments were reviewed and approved by the Ethics Committee of the Second Xiangya Hospital, Central South University (Animal Permit No. 2022,123, Human samples Permit No.2022,593). All the procedures conform to the Guide for the Care and Use of Laboratory Animals, NIH publication, 8th edition, 2011. The human samples conformed to the principles outlined in the Declaration of Helsinki.

### Human samples

A total of 5 pairs of radial artery segments from patients with CRF and normal donors (ND) were collected from The Second Xiangya Hospital of Central South University. CRF radial arteries were obtained from CRF patients who required arteriovenous fistula angioplasty. ND radial arteries were obtained from patients who underwent arm amputation due to trauma in the Department of Orthopedics in our hospital. The inclusion and exclusion criteria were as follows: CRF patients with the glomerular filtration rate (GFR) < 15 mL/min/1.73 m^2^ of body-surface areas and no history of AIDS, diabetes and malignant tumors were selected as the CRF group. The selected normal donors were age-matched, had normal renal function, and had no other diseases such as malignant tumors, hypertension, diabetes, coronary heart disease, etc. All participants signed written informed consent.

### Cell culture and transfection

Mice AFs (1-3111) and VSMCs (1-3027) were purchased from CHI Scientific, Inc. They were both cultured in DMEM: F12 (1:1) medium (C11330500BT, Gibco, Invitrogen, New York, USA) supplemented with 10% fetal bovine serum (FBS) (1907B, Bovogen, New Zealand, Australia) and 1% penicillin/streptomycin (03-031-1BCS, Gibco, Invitrogen, New York, USA). Cells were incubated at 37 °C in a humidified atmosphere containing 5% CO_2_. The culture medium was refreshed every 2 days. The AFs and VSMCs at passage 3–5 (P3-5) were used for subsequent experiments. To establish the calcification model, the VSMCs were incubated with the medium containing 3.5 mM inorganic phosphorus (NaH_2_PO_4_: Na_2_HPO_4_ = 1:2, pH = 7.0. Na_2_HPO_4_, S5136, Sigma-Aldrich, Saint Louis, USA; NaH_2_PO_4_, S5011, Sigma-Aldrich, Saint Louis, USA), and the medium containing 0.9 mM inorganic phosphorus was used as the control. For the treatment of exosomes, when the confluence density of VSMCs reached about 70%, the medium containing 0.9 mM or 3.5 mM inorganic phosphorus was added, and AFs-Exos (200 µg/mL) were used for treatment. For cell transfection, miR-21-5p mimics, miR-21-5p inhibitor, Crim1 siRNA, and their respective control oligos (100 nM) were transfected into VSMCs using siRNA mate according to the manufacturer’s instructions. miR-21-5p mimics (miR10000530), inhibitor (miR20000530) and their control oligos were purchased from Ribobio (Guangzhou, China). Crim1 siRNA, control oligos and siRNA mate (G04003) were purchased from GenePharma (Shanghai, China).

### Exosome isolation and identification

AFs were cultured with the exosome-depleted medium containing 0.9 mM inorganic phosphorus or 3.5 mM inorganic phosphorus for 48 h, and then the cell supernatant was collected. Exosomes from AFs supernatant were isolated by differential centrifugation. Briefly, the supernatant was centrifuged at 300 g for 10 min, 2000 g for 10 min, 10,000 g for 30 min at 4 °C, and ultracentrifuged at 100,000 g for 70 min to sediment the pellets. The pellets were then re-suspended in PBS and ultracentrifuged at 100,000 g for 70 min at 4 °C. The exosomes-enriched pellet was re-suspended in the appropriate volume of PBS and filtered through 0.22 μm filters (SLGPR33RB, Millipore, USA) to obtain the sterile exosome suspension. Aliquot 5 µL of exosome suspension and the protein quantification of exosomes was measured by the BCA kit (CW0014S, cwbiotech, Beijing, China).

For the identification of exosomes, transmission electron microscopy (TEM) (Hitachi, Tokyo, Japan) was used to observe the morphology and size of exosomes. The diameter size distribution of exosomes was analyzed by dynamic light scattering (DLS) with a Zetasizer Nano ZS instrument (Malvern Instruments). Western blot was used to detect the expression of exosomal marker proteins TSG101, CD9, and CD81.

### Exosomes uptake by VSMCs

To confirm that the AFs-Exos could be taken up by VSMCs, we labeled the exosomes with a red fluorescent dye (PKH26, MINI26-1KT, Sigma), and co-incubated the labeled exosomes with VSMCs as described previously [36]. Briefly, 4 µL of PKH26 dye was first dissolved in 500 µL Dilute C solution. 100 µg exosomes were added to the mixture and incubated at room temperature for 5 min. Then 500 µL BSA was added to terminate the reaction. The unbound dyes were removed by ultracentrifugation at 100, 000 g for 70 min. The labeled exosomes were co-incubated with VSMCs at 37℃ for 12 h. After washing with PBS, VSMCs were fixed with 4% paraformaldehyde at room temperature for 30 min. After washing with PBS, VSMCs were incubated with DAPI (C1005, Beyotime Biotechnology, Shanghai, China) for 5 min at room temperature to stain nuclei. After washing with PBS, the red fluorescent signals in VSMCs were detected by the fluorescence microscope.

### Western blot analysis

The protein expression was determined by Western blotting as previously described [37]. 30 µg protein extracts were separated by SDS-PAGE and transferred to polyvinylidene fluoride membranes (IPVH00010, PVDF, Millipore, Billerica, MA). After blocking with 5% non-fat milk for 1 h, the membrane was incubated overnight at 4 °C with primary antibody, including CD9 (ab92726, 1:1000, abcam), CD81 (ab109201, 1:1000, abcam), TSG101 (bs-1365R, 1:1000, bioss), Runx2 (ab23981, 1:2000, abcam), BMP2 (bs-10696R, 1:1000, bioss), GAPDH (10494-1-AP, 1:4000, proteintech), BMP4 (bs-1374R, 1:1000, bioss), Crim1 (bs-21654R, 1:1000, bioss), followed by incubation with the horseradish peroxidase-conjugated secondary antibody for 1 h at room temperature. The immunoreactive bands were visualized by the enhanced chemiluminescence reagent (WBKLS0100, Millipore, Billerica, MA) and imaged by Amersham Imager 600 analyser (General Electric, USA).

### Alizarin Red S staining

VSMCs were seeded in 24-well plates and treated with the conditioned medium of AFs or AFs-Exos for at least 21 days. After washing with PBS, VSMCs were fixed with 4% paraformaldehyde for 30 min at room temperature, washed three times with PBS, and stained with Alizarin Red staining solution (CR2203058, Servicebio, Wuhan, China) for 5 min at room temperature. After washing with PBS, cells were observed and photographed under a microscope.

### Alkaline phosphatase (ALP) staining and ALP activity assay

For the ALP staining of VSMCs, cells were seeded in 24-well plates and treated with conditioned medium of AFs or AFs-Exos for 14 days. Then VSMCs were washed with PBS, fixed with 4% paraformaldehyde at room temperature for 30 min, and washed 3 times with PBS. The VSMCs were incubated with ALP staining working solution (C3206, Beyotime Biotechnology, Shanghai, China) at room temperature for 5–30 min in the dark according to the manufacturer’s instructions. After washing with PBS, VSMCs were photographed under a microscope.

The ALP activity was measured by an ALP activity assay kit (A059-2-2, njjcbio, Nanjing, China). The VSMCs were washed three times with PBS, and the total cellular proteins were extracted. The samples and reagents were added to a 96-well plate according to the manufacturer’s instructions. After incubation at 37 °C for 15 min, the chromogenic reagent was added. The absorbance was observed at 520 nm by using a microplate reader. ALP activity was normalized by the total cellular protein concentration.

### Transwell co-culture system

Transwell co-culture experiments were performed using 6-well transwell inserts (3450, Corning, NY, USA) with 0.4 μm pore-sized filters as described previously [2]. AFs (1 × 10^5^ cells per well) were first seeded into the upper chamber and pretreated with or without GW4869 (UR21021, umibio, Shanghai, China) for 48 h. VSMCs (2 × 10^5^ cells per well) were seeded into the lower chamber of the 6-well plate. Both AFs and VSMCs were cultured with the exosome-depleted medium containing 0.9 mM inorganic phosphorus or 3.5 mM inorganic phosphorus. After 72 h, the total cellular proteins of VSMCs in the lower chamber were collected for detection.

### qRT-PCR analysis

RNA was extracted from exosomes using the miRNeasy® Mini kit (217084, Qiagen) as the manufacturer’s instructions described. Cellular total RNA was extracted using TRIzol Reagent (15596026, Invitrogen, Carlsbad, USA) as described previously [38]. According to the manufacturer’s instructions, Mir-X miRNA First-Strand Synthesis Kit (638315, Takara, Japan) was used for reverse transcription and qRT-PCR amplification of RNA extracted from exosomes and cells. The sequences of miRNAs were as follows: mmu-miR-122-5p (5’-UGGAGUGUGACAAUGGUGUUUG-3’), mmu-miR-155-5p (5’-UUAAUGCUAAUUGUGAUAGGGGU-3’), mmu-miR-29a-3p (5’-UAGCACCAUCUGAAAUCGGUUA-3’), mmu-miR-487b-3p (5’-AAUCGUACAGGGUCAUCCACUU-3’), mmu-miR-21-5p (5’-UAGCUUAUCAGACUGAUGUUGA-3’), mmu-miR-135a-5p (5’-UAUGGCUUUUUAUUCCUAUGUGA-3’), mmu-miR-124-3p (5’- UAAGGCACGCGGUGAAUGCC-3’), mmu-miR-125b-5p (5’-UCCCUGAGACCCUAACUUGUGA-3’). The following 5’ miRNA specific primers were purchased from GeneCopoeia: mmu-miR-122-5p (MmiRQP0056), mmu-miR-155-5p (MmiRQP0890), mmu-miR-29a-3p (MmiRQP0371), mmu-miR-487b-3p (MmiRQP0525), mmu-miR-21-5p (MmiRQP0316), mmu-miR-135a-5p (MmiRQP0170), mmu-miR-124-3p (MmiRQP0074), mmu-miR-125b-5p (MmiRQP0096). Relative quantification was calculated using the 2^–ΔΔCT^ method and U6 small nuclear RNA was served as the reference for normalization.

### Enzyme digestion experiment

For the Rnase-treated group, exosomes were treated with 100 µg/mL Rnase A (10405ES03, Yeasen Biotech, Shanghai, China) at 37 °C for 15 min. For the ProtK + Rnase-treated group, exosomes were first treated with 100 µg/mL proteinase K (ProtK) (39450-01-6, Sigma-Aldrich, Saint Louis, USA) for 30 min at 37 °C, followed by Rnase A treatment. For the Triton X + Rnase-treated group, exosomes were first treated with 10% Triton X-100 (T8200, Solarbio, Beijing, China) at room temperature for 30 min, followed by Rnase A treatment. The control group was left untreated. The miRNAs in the treated exosomes were extracted, and the expression level of miR-21-5p was detected by Quantitative real-time PCR (qRT-PCR).

### Plasmid constructs and luciferase reporter assay

Wild-type and mutant segments of the Crim1 3’-UTR, including predicted miR-21-5p binding sites, were cloned into the PmeI and XbaI restriction sites of pGL3 luciferase reporter vector (E1330, Promega). Mutant Crim1 3’-UTR was constructed using the QuikChange Site-Directed Mutagenesis Kit (Stratagene).

VSMCs were co-transfected with a luciferase reporter carrying wild-type or mutant Crim1 3’-UTR, and miR-21-5p mimics or mimics control. 48 h after transfection, the luciferase activity was detected with a luciferase assay system (Promega, USA). The renilla luciferase activity was used to normalize the relative luciferase activity of cells.

### Animals

6- to 8-week-old male C57BL/6J mice (weighing 20–25 g) were purchased from SJA Laboratory Animal Co., Ltd (Hunan, China) and housed in standard cages on a 12 h light/dark cycle. We established the mouse model of CRF with vascular calcification using the 5/6 nephrectomy (5/6 NTP) plus a high-phosphorus diet (0.9% Pi) feeding method as described in our previous studies [2, 39, 40]. To study the role of AFs^HPi^-Exos in the development of vascular calcification in mice with CRF, the mice were randomly divided into four treatment groups (n = 5 per group): Sham group (Sham-operated group fed with high phosphorus diet only), 5/6 NTP group (5/6 nephrectomy plus high phosphorus diet feeding group), 5/6 NTP + AFs^NPi^-Exos group (5/6 NTP mice were intravenously injected with 100 µg AFs^NPi^-Exos), 5/6 NTP + AFs^HPi^-Exos group (5/6 NTP mice were intravenously injected with 100 µg AFs^HPi^-Exos). In addition, to examine the role of miR-21-5p in high phosphorus-induced vascular calcification in CRF mice, the mice were randomly divided into the sham group and 5/6 NTP group. The mice in the sham group were intravenously injected with AFs^ctrl^-Exos. The 5/6 NTP mice were randomly divided into three treatment groups (n = 5 per group): AFs^ctrl^-Exos group, AFs^ctrl M^-Exos group, AFs^miR−21 M^-Exos group (100 µg exosomes per mice).

The 5/6 NTP mouse model was established as follows, mice were anesthetized by intraperitoneal injection of 1% pentobarbital sodium (60 mg/kg). The upper and lower poles of the left kidney were removed first, and the adrenal glands were preserved. A total right nephrectomy was performed one week later. In the sham-operated group, after exposure of the kidney, the kidney capsule was peeled off without removing the kidney, and then the skin was closed. One week after the operation, the sham mice and 5/6 NTP mice were fed a diet containing 0.9% phosphorus for 4 weeks to accelerate the vascular calcification of the mice. During the high-phosphorus feeding period, vehicles or exosomes (100 µg exosomes in 100 µL PBS) were intravenously injected into the mice every other day for a total of 4 weeks (fourteen injections in total). The mouse model of CRF with vascular calcification was set up successfully, which was confirmed by the increases in the level of serum urea nitrogen, creatinine, calcium and phosphorus. The thoracic aortas of mice were removed for the detection of calcification markers.

### Tracing of exosomes in vivo

The exosomes secreted by HPi-induced AFs were labeled with DiR dye (D12731, Invitrogen, USA) according to the manufacturer’s instructions and injected into the 5/6 NTP-induced C57BL/6J mice by tail vein. In detail, the DiR solution was diluted with ethanol to obtain 200 µg/mL working solution. 5 µL DiR working solution was added to 100 µg exosomes and incubated in the dark for 1 h at room temperature. The unbound dyes and ethanol were removed by ultracentrifugation at 100,000 g at 4℃ for 70 min, and the precipitate was re-suspended in PBS at a concentration of 100 µg exosomes/100 µL PBS. DiR-labeled exosomes, PBS or DiR (100 µL per mice) were injected into the tail vein of 5/6 NTP mice (n = 5 per group). In vivo fluorescence imaging was performed within 12 h, and organs were also taken for imaging.

### Measurement of arterial calcification

After the artery samples were collected, we first removed the vascular branches and adipose tissues around the arteries. Then the arteries were fixed with 4% paraformaldehyde, embedded in paraffin and cut into 5 μm sections. Paraffin sections were dewaxed and dehydrated with turpentine and a series of graded ethanol.

For Alizarin Red S staining, the sections were washed with PBS, and then incubated with Alizarin Red staining solution at room temperature for 15 s. The sections were observed under a microscope and photographed.

For Von Kossa staining, after washing with double-distilled water, the sections were incubated with Von Kossa staining solution (G1043, Servicebio, Wuhan, China) under ultraviolet light for 2 h. After the sections were thoroughly washed with double-distilled water, the nucleus was stained with hematoxylin, and the cytoplasm was stained with eosin. Subsequently, the sections were dehydrated in ethanol, and sealed with neutral gum. Photographs were taken under the microscope and positive staining areas were analyzed with Image J software.

For the detection of ALP activity and calcium content in aortic tissues, tissue homogenate supernatant was prepared first. RIPA lysate was added to the arterial tissue and ground in the grinding machine (frequency 70 Hz, grinding time 60 s, pausing time 20 s, cycle 3 times), followed by ultrasonic lysis and centrifugation. The supernatant was taken as the tissue homogenate supernatant, and the total protein concentration of tissues was measured with a BCA kit. The ALP activity assay method was the same as described above. Calcium content was determined by using a commercial kit (C004-2-1, njjcbio, Nanjing, China) according to the manufacturer’s instructions. The ALP activity and calcium content were normalized with total protein levels.

### Immunohistochemistry and immunofluorescence

For immunohistochemistry, sections of the arteries were dewaxed and dehydrated as described previously. Immunohistochemical analysis was performed using Universal Two-step Immunohistochemistry Kit (PV-9000, zsbio, Beijing, China) according to the manufacturer’s instructions. Briefly, antigens were retrieved by trypsin, endogenous peroxidase was removed by 3% hydrogen peroxide, followed by 5% BSA blocking for 1 h. Sections were then incubated overnight at 4 °C with primary antibody for Runx2 (bs-1134R, 1:250, bioss), BMP2 (bs-10696R, 1:250, bioss), BMP4 (bs-1374R, 1:250, bioss), Crim1 (bs-21654R, 1:250, bioss). The next day, sections were incubated with reaction enhancer and secondary antibody, and positive areas were detected with DAB chromogenic solution. Finally, nuclei were stained with hematoxylin.

For immunofluorescence, frozen sections of aortas were incubated with the exosomal marker TSG101 rabbit antibody (bs-1365R, 1:200, bioss) and VSMC marker α-SMA mouse antibody (GB13044, 1:200, Servicebio). Subsequently, the binding primary antibodies was visualized using FITC/Cy3-conjugated secondary antibody. The nuclei were stained with DAPI. Sections were observed and photographed under a fluorescence microscope (Nikon Instruments Korea, Seoul, Korea).

### Statistical analysis

All data are presented as mean ± SD. Statistical analysis was performed with the GraphPad Prism version 8.0 software. Student’s *t*-test was conducted for comparisons between two groups. One-way ANOVA followed by Dunnett’s test was used for comparisons between more than two groups. *P* < 0.05 was considered to be statistically significant. All experiments were repeated at least three times, and representative experimental results are shown in the figures.

## Results

### Artery calcification in CRF patients

Typical vessel wall media calcification often occurs in patients with CRF [41–43]. We collected radial arteries from ND and CRF patients and compared their calcification levels. Alizarin Red S and Von Kossa staining showed increased mineral deposition in the radial artery of CRF patients compared with normal donors (Additional file 1: Fig. S1A). Immunohistochemical staining showed that the expression of Runx2 and BMP2 was increased in the CRF group (Additional file 1: Fig. S1B). These results confirm that the arteries of CRF patients are severely calcified. Therefore, we further explored the specific mechanism of CRF-induced arterial calcification.

### Conditioned medium of HPi-induced AFs promotes VSMCs calcification

**Fig. 1:**
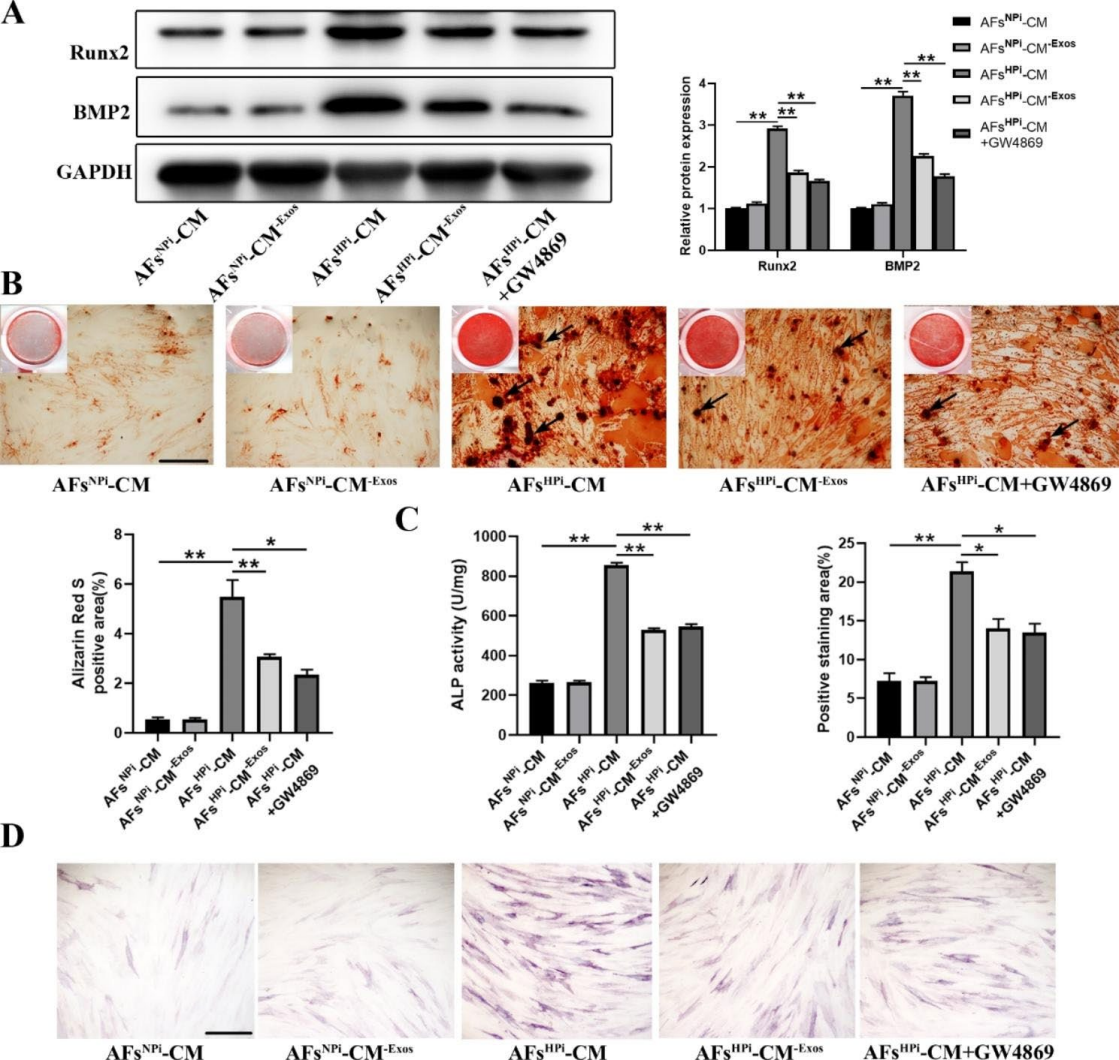
The conditioned medium of HPi-induced AFs promotes VSMCs calcification. VSMCs were treated with 0.9 mM or 3.5 mM inorganic phosphate-induced AFs conditioned medium (AFs^NPi^-CM, AFs^HPi^-CM), 0.9 mM or 3.5 mM inorganic phosphate-induced AFs exosomes-free conditioned medium (AFs^NPi^-CM^− Exos^, AFs^HPi^-CM^− Exos^), 3.5 mM inorganic phosphate-induced GW4869-pretreated AFs conditioned medium (AFs^HPi^-CM + GW4869). **A** Western blot and quantitative analysis of Runx2 and BMP2 protein levels in VSMCs. **B** The formation of mineralized nodules in VSMCs was detected by Alizarin red staining. The black arrows indicate mineralized nodules in VSMCs. Representative microscopic views are shown. The scale bar represents 500 μm. **C** ALP activity in VSMCs was measured by the ALP kit. **D** ALP staining and quantitative analysis of the percentage of ALP staining positive cells in total area. Representative microscopic views are shown. The scale bar represents 500 μm. Three independent experiments were performed, and representative data are shown. Data are shown as mean ± SD. *p < 0.05, **p < 0.01

Hyperphosphatemia plays an important role in promoting the development of vascular calcification in patients with CRF [16]. In vivo, both AFs and VSMCs can be stimulated by high phosphorus when the vessel wall is in a high phosphorus microenvironment. To investigate the effects of the conditioned medium of HPi-induced AFs on VSMCs calcification, we treated AFs with inorganic phosphorus at a concentration of 0.9 mM or 3.5 mM for 48 h and then collected the supernatant (AFs^NPi^-CM, AFs^HPi^-CM) as conditioned medium to culture VSMCs. We found that compared with AFs^NPi^-CM, AFs^HPi^-CM significantly promoted the expression of osteogenic differentiation proteins Runx2 and BMP2 in VSMCs (Fig. 1A). Moreover, Alizarin red staining showed that AFs^HPi^-CM significantly increased the formation of mineralized nodules (Fig. 1B). AFs^HPi^-CM also caused a remarkable increase in ALP activity and ALP positive staining area compared to the AFs^NPi^-CM group, as evidenced by the ALP activity assay and ALP staining (Fig. 1C-D).

To explore whether the effects of AFs^HPi^-CM on VSMCs calcification were mediated by exosomes, we removed exosomes in the conditioned medium of AFs by ultracentrifugation (AFs^NPi^-CM^− Exos^ and AFs^HPi^-CM^− Exos^). The results showed that AFs^HPi^-CM induced increases in the protein expression of Runx2 and BMP2, the formation of mineralized nodules, ALP activity, and ALP positive staining area in VSMCs. However, after we removed exosomes from the AFs^HPi^-CM by ultracentrifugation, the pro-calcification effect was significantly attenuated (Fig. 1A-D). In addition, AFs were pretreated with GW4869 for 48 h to inhibit the biogenesis or release of exosomes. We found that pretreatment of GW4869 abolished the pro-calcification effect of AFs^HPi^-CM on VSMCs (Fig. 1A-D). These results indicate that the Exos derived from high phosphorus-induced AFs are essential in promoting the calcification of VSMCs.

#### Identification and uptake assay of exosomes

**Fig. 2:**
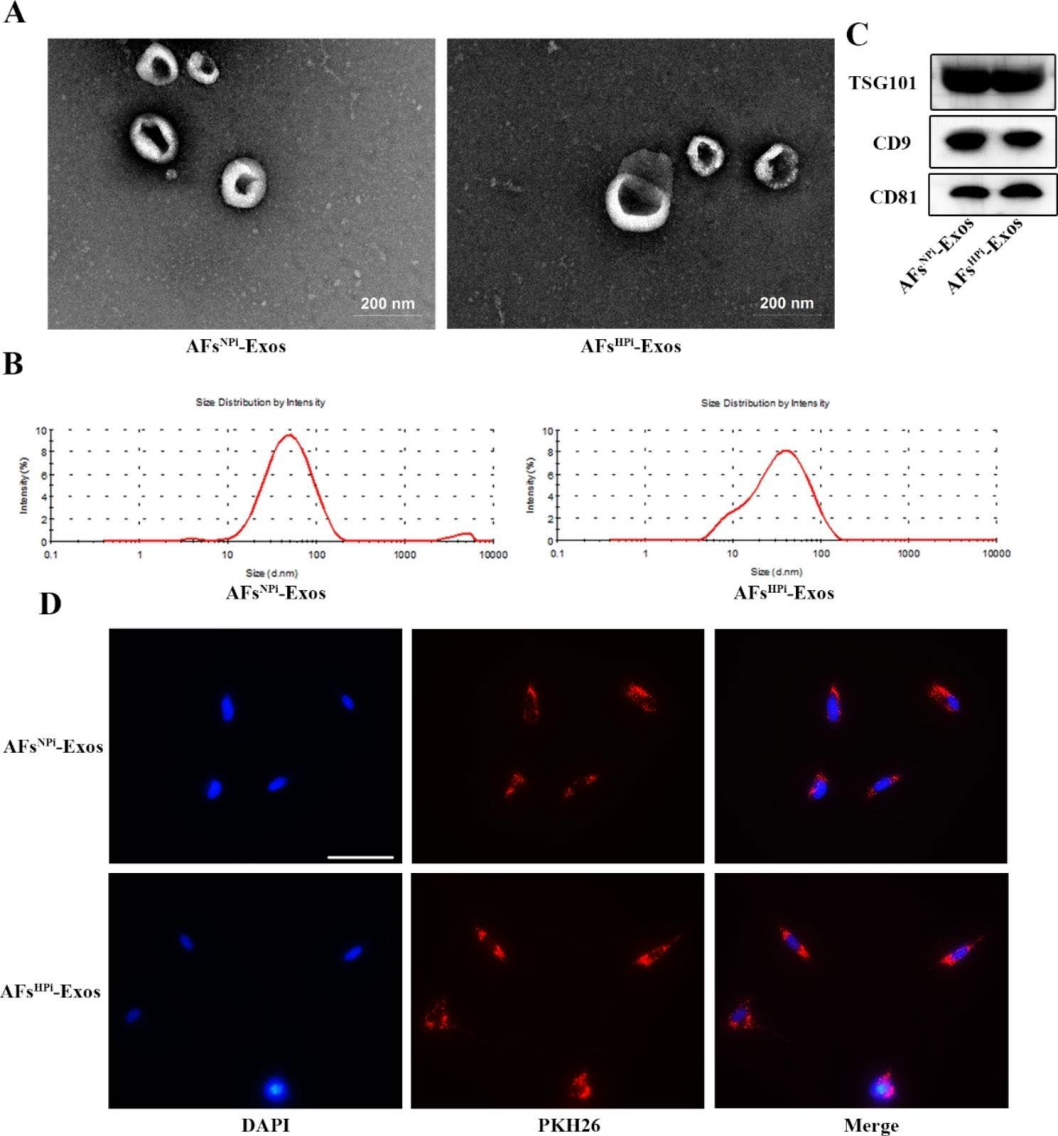
Identification and uptake assay of AFs-derived exosomes. **A** Representative transmission electron microscopic photographs of AFs^NPi^-Exos and AFs^HPi^-Exos. The scale bar represents 200 μm. **B** The diameter distribution of AFs^NPi^-Exos and AFs^HPi^-Exos was detected by DLS analysis. **C** Western blot analysis of exosome-specific markers TSG101, CD9, and CD81 in exosomes secreted by NPi/HPi-induced AFs (AFs^NPi^-Exos, AFs^HPi^-Exos). **D** Representative images of AFs^NPi^-Exos and AFs^HPi^-Exos uptake by VSMCs after 12 h incubation. Nuclei stained by DAPI in blue and AFs-Exos stained by PKH26 in red. Scale bar: 50 μm

We isolated exosomes from the conditioned medium of NPi or HPi-induced AFs (AFs^NPi^-Exos, AFs^HPi^-Exos) by differential centrifugation and identified them by TEM, particle size analysis, and western blot. TEM revealed that AFs^NPi^-Exos and AFs^HPi^-Exos exhibited round, cup-shaped morphology (Fig. 2A). The majority of AFs^NPi^-Exos and AFs^HPi^-Exos had an average diameter distribution of 50–150 nm as shown by DLS measurement (Fig. 2B). Exosome-specific markers TSG101, CD9, and CD81 were both expressed in AFs^NPi^-Exos and AFs^HPi^-Exos (Fig. 2C). These data suggest that these nanoparticles were actually exosomes. To investigate whether AFs-derived exosomes could be taken up by VSMCs, exosomes were labeled with red fluorescent dye PKH26 and incubated with VSMCs for 12 h. The results revealed that both the labeled AFs^NPi^-Exos and AFs^HPi^-Exos successfully entered into the perinuclear region of VSMCs (Fig. 2D).

### Exosomes derived from AFs induced by high phosphorus promote VSMCs calcification

**Fig. 3:**
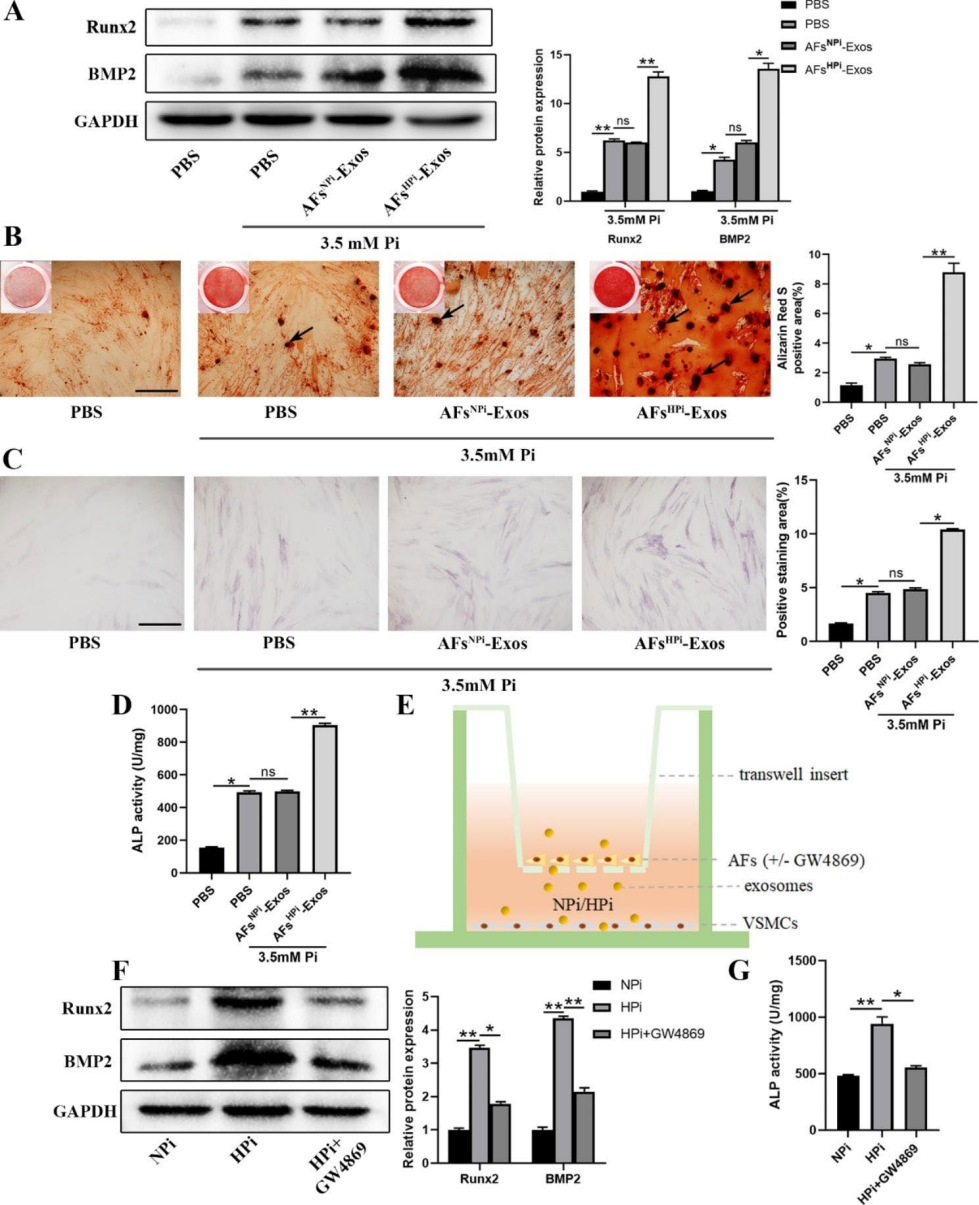
Exosomes derived from AFs induced by high phosphorus promote VSMCs calcification. **A** Western blot was used to determine the effect of AFs^NPi^-Exos or AFs^HPi^-Exos treatment on Runx2 and BMP2 protein levels in 3.5 mM Pi-induced VSMCs. VSMCs treated with 0.9 mM Pi plus PBS were used as control. **B** Representative images and quantitative analysis of Alizarin red staining in 3.5 mM Pi-induced VSMCs cultured with AFs^NPi^-Exos or AFs^HPi^-Exos. The black arrows indicate mineralized nodules in VSMCs. The scale bar represents 500 μm. ALP staining (**C**) and ALP activity (**D**) of AFs^NPi^-Exos or AFs^HPi^-Exos treated VSMCs under 3.5 mM Pi treatment. Representative micrographs are shown. The scale bar represents 500 μm. **E** The transwell co-culture system was used to detect the calcification of VSMCs co-cultured with AFs pretreated with or without GW4869 under the treatment of NPi or HPi for 3 days. Western blot analysis of Runx2 and BMP2 protein levels (**F**) and ALP activity (**G**) in VSMCs co-cultured with AFs. Data are presented as mean ± SD from triplicate experiments. *p < 0.05, **p < 0.01

To determine the direct effects of AFs-Exos on VSMCs calcification, we first treated VSMCs with 3.5 mM inorganic phosphorus (HPi) to establish the calcification model of VSMCs, and incubated VSMCs with 200 µg/mL exosomes isolated from AFs induced by 0.9 mM inorganic phosphorus (NPi) or HPi. In the control group, the VSMCs were treated with NPi plus PBS. The results showed that compared with the control group, HPi treatment enhanced the protein expression of Runx2 and BMP2, increased the mineralized nodules formation, ALP positive staining area, and ALP activity in VSMCs, indicating that the calcification model of VSMCs was successfully established. Furthermore, these pro-calcification effects were enhanced by AFs^HPi^-Exos (Fig. 3A-D).

Next, a transwell co-culture system was used to detect the crosstalk between AFs and VSMCs under the high phosphorus microenvironment. AFs pretreated with or without GW4869 were co-cultured with VSMCs in normal or high phosphorus systems (Fig. 3E). We found that compared with the control group, the expression of Runx2 and BMP2 proteins and the ALP activity in VSMCs co-cultured with high phosphorus-treated AFs were significantly increased, however, GW4869 pretreatment significantly attenuated this effect (Fig. 3F-G).

Taken together, these results suggest that exosomes released from HPi-induced AFs are necessary for the calcification of VSMCs.

#### AFs^HPi^-Exos enrich and transfer miR-21-5p

**Fig. 4:**
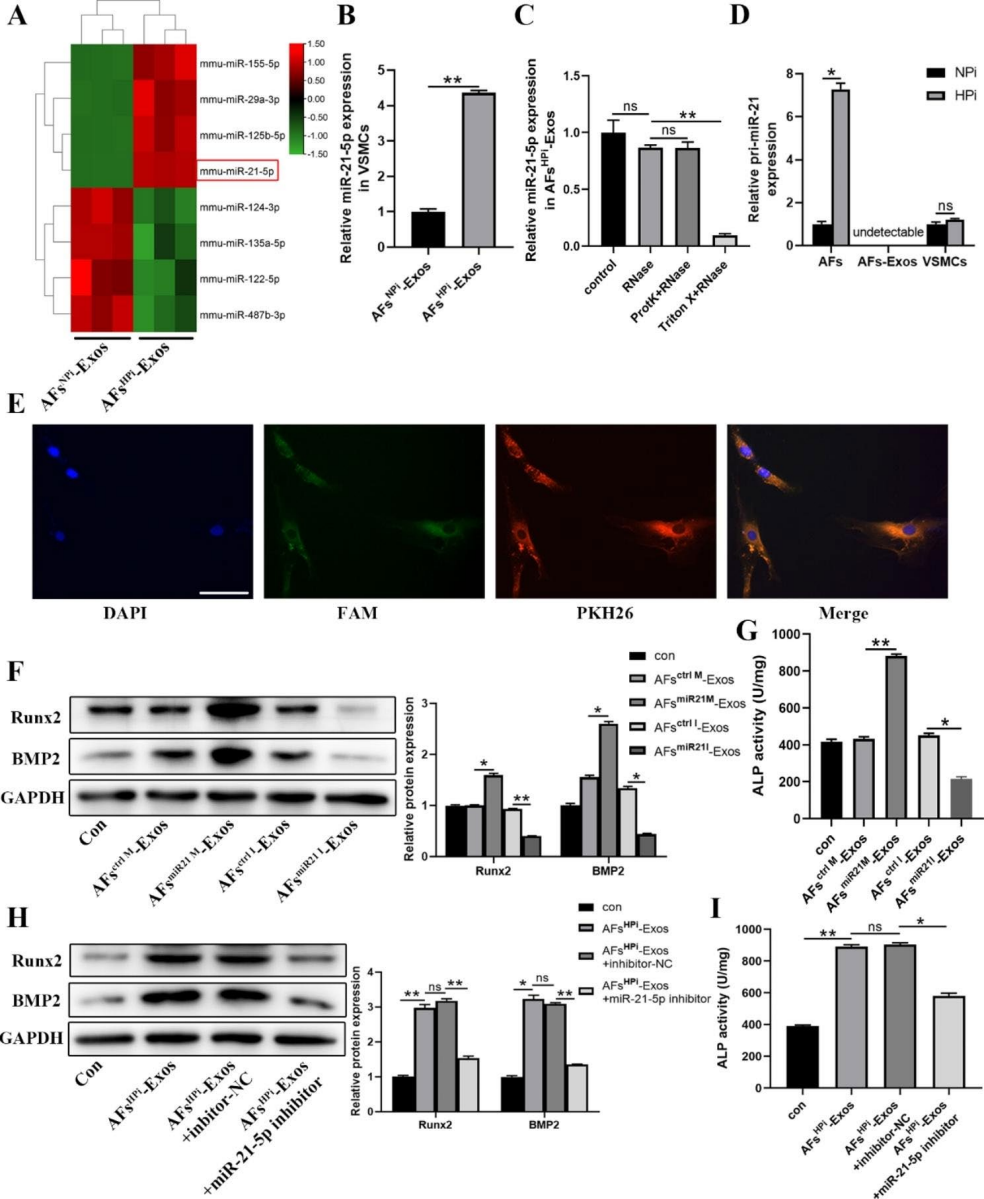
AFs^HPi^-Exos transport miR-21-5p to VSMCs and promote the calcification of VSMCs. **A** Heatmap showing expression profiles of candidate miRNAs in AFs^NPi^-Exos and AFs^HPi^-Exos. **B** qPCR analysis showing the level of miR-21-5p in VSMCs treated with AFs^NPi^-Exos or AFs^HPi^-Exos. **C** The miR-21-5p levels in AFs^HPi^-Exos treated with RNase, proteinase K (ProtK), or Triton X-100 as measured by qPCR. **D** pri-miR-21 levels in NPi/HPi treated AFs, AFs^NPi^-Exos/AFs^HPi^-Exos and AFs^NPi^-Exos/AFs^HPi^-Exos treated VSMCs as measured by qPCR. **E** Fluorescence microscopy analysis of PKH26-labeled exosomes derived from FAM-miR-21-5p-mimics transfected AFs internalization by VSMCs. FAM-miR-21-5p signals (green) were co-localized with PKH26 signals in the perinuclear region of VSMCs (PKH26 in red and DAPI in blue). The scale bar represents 50 μm. Western blot (**F**) and ALP activity assay (**G**) showing the Runx2 and BMP2 protein expression and ALP activity in VSMCs after treatment with vehicle or exosomes derived from AFs transfected with miR-21-5p mimics/miR-21-5p inhibitor/negative controls. The levels of Runx2 and BMP2 (**H**) and ALP activity (**I**) in VSMCs after miR-21-5p inhibitor transfection under AFs^HPi^-Exos treatment. The VSMCs in all groups were induced by 3.5 mM Pi simultaneously. Data are the mean ± SD of three independent experiments. *p < 0.05, **p < 0.01

We further explored the functional molecules that mediate the pro-calcification effect of AFs^HPi^-Exos. We first selected eight miRNAs that have been reported to be highly expressed in fibroblasts and associated with osteogenic differentiation [44–60]. qRT-PCR analysis was used to detect the expression levels of 8 miRNAs in AFs^NPi^-Exos and AFs^HPi^-Exos. We displayed the results of qRT-PCR in a heatmap. The results showed that miR-21-5p was most significantly elevated in AFs^HPi^-Exos (Fig. 4A). Furthermore, compared with AFs^NPi^-Exos, AFs^HPi^-Exos treatment significantly increased the miR-21-5p level in VSMCs (Fig. 4B). We also detected the basal expression of miR-21-5p. The results showed that miR-21-5p was expressed in both AFs and VSMCs, and the miR-21-5p expression level in AFs was about 4 times that of VSMCs (Additional file 1: Fig. S2), which provided an important premise for the role of the abundant exogenous miR-21-5p transported by AFs-Exos in VSMCs. Previous studies indicated that miR-21-5p promoted the osteogenic differentiation of bone marrow mesenchymal stromal cells (BMSCs) and hFOB1.19 cells [52, 61]. Therefore, we finally selected miR-21-5p for further study.

In order to confirm that miR-21-5p could be protected by exosomal bilayer membrane structure and that the increased miR-21-5p was carried by AFs^HPi^-Exos, rather than protein-bound free RNA, we treated AFs^HPi^-Exos with Triton X-100, proteinase K (ProtK) or Rnase. In the control group, AFs^HPi^-Exos were treated with equal volume of vehicle. The results showed that compared with the control group, the levels of miR-21-5p in AFs^HPi^-Exos in the Rnase group and the ProtK + Rnase group did not change significantly, while in the Triton X + Rnase group, after the integrity of exosomal membrane structure was disrupted by Triton X-100, Rnase treatment could significantly reduce the level of miR-21-5p (Fig. 4C). These results suggest that the membrane structure of exosomes protects miR-21-5p from degradation by proteinase and Rnase. To verify that AFs^HPi^-Exos can transfer mature miR-21-5p from AFs to VSMCs, we examined the expression of primary (pri)-miR-21 (the precursor of miR-21-5p). We found that pri-miR-21 only exhibited higher levels in AFs^HPi^, while it was not detectable in exosomes. Moreover, AFs^HPi^-Exos treatment did not alter the level of pri-miR-21 in VSMCs (Fig. 4D), indicating that the elevated level of miR-21-5p in VSMCs was not derived from their own synthesis, but from the transfer of AFs^HPi^-Exos.

To further verify that exosomal miR-21-5p could be taken up by VSMCs, we transfected AFs with miR-21-5p mimics/inhibitor. In order to ensure the success of transfection, we first examined the transfection efficiency. The results showed that compared with the control group, transfection of miR-21-5p mimics significantly increased the level of miR-21-5p in AFs and their secreted exosomes, whereas miR-21-5p inhibitor exerted the opposite effect (Additional file 1: Fig. S3A-B). Next, we labeled the exosomes derived from FAM-miR-21-5p mimics transfected AFs with PKH26 and incubated VSMCs with the labeled exosomes. We observed the co-localization of green and red fluorescence signals in the cytoplasm of VSMCs (Fig. 4E), suggesting that exosomal miR-21-5p was internalized by VSMCs.

These data suggest that miR-21-5p is enriched in AFs^HPi^-Exos and can be transferred by exosomes from AFs to VSMCs.

#### HPi-induced exosomal miR-21-5p promotes VSMCs calcification

To determine the role of miR-21-5p in the exosome-induced regulation of VSMCs calcification, VSMCs were cultured with exosomes derived from miR-21-5p mimics/inhibitor transfected AFs under 3.5 mM Pi treatment. We found that AFs^miR21M^-Exos could significantly enhance the Runx2, BMP2 protein expression, and ALP activity in VSMCs, while AFs^miR21I^-Exos had the opposite effects (Fig. 4F-G). Then VSMCs incubated with AFs^HPi^-Exos were additionally treated with miR-21-5p inhibitor (a specific inhibitor targeting miR-21-5p). The results showed that the pro-calcification effect of AFs^HPi^-Exos was reversed by miR-21-5p inhibitor (Fig. 4H-I). These data suggest that AFs-Exos regulated the calcification of VSMCs by transferring miR-21-5p.

#### MiR-21-5p promotes VSMCs calcification by targeting the Crim1 gene

**Fig. 5:**
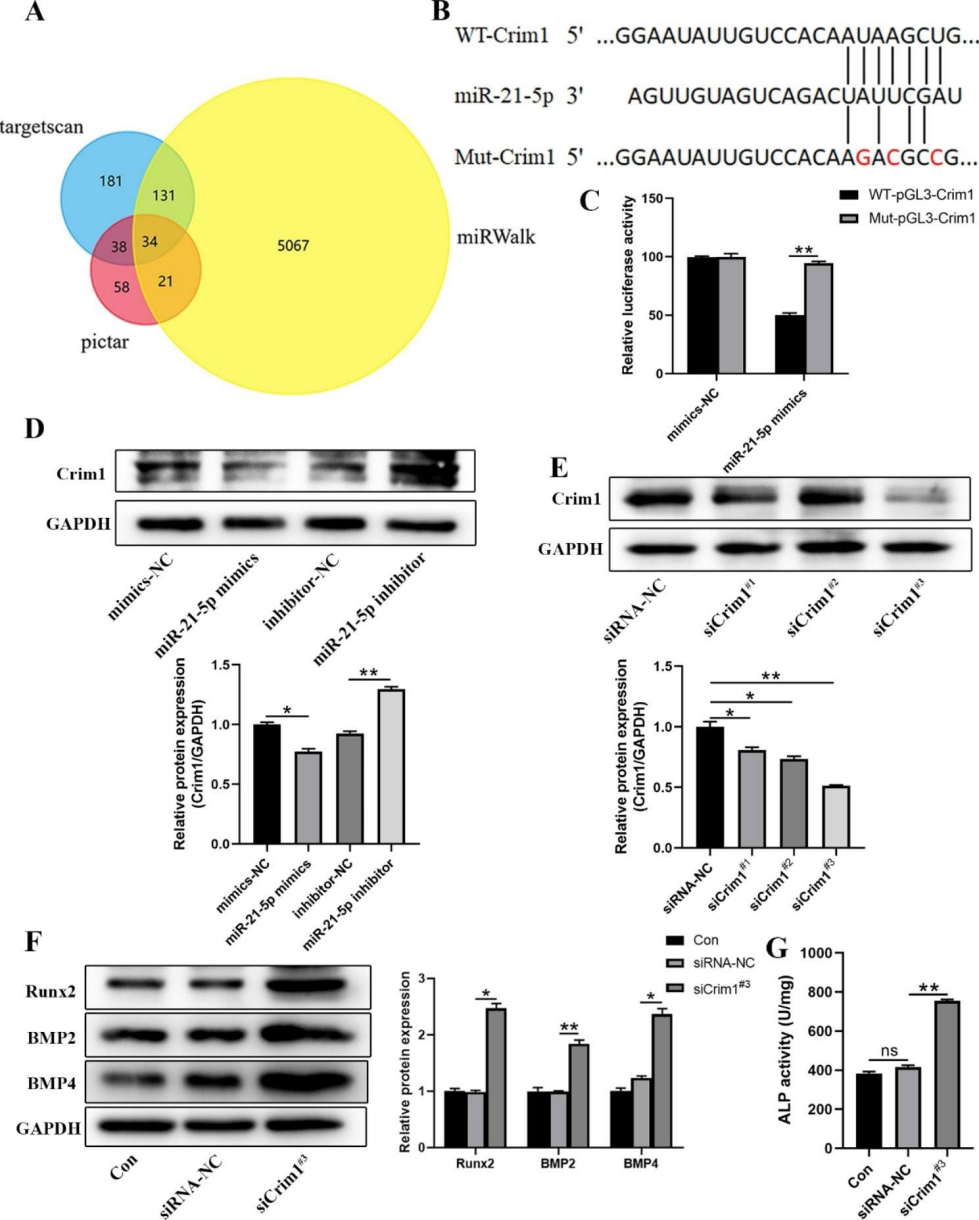
miR-21-5p promotes VSMCs calcification by targeting the Crim1 gene. **A** Venn diagram showing potential target genes of miR-21-5p predicted by targetscan, pictar, miRWalk databases. **B** Schematic illustration of the sequences for miR-21-5p and the WT or mutated 3’-UTR of Crim1 mRNA. **C** Luciferase reporter assays showing the relative luciferase activity of Crim1 WT or mutated 3’-UTR reporter plasmids in VSMCs co-transfected with miR-21-5p mimics or mimics-NC. Firefly luciferase activity was normalized to renilla luciferase activity. **D** Western blot analysis of Crim1 levels in VSMCs transfected with miR-21-5p mimics, miR-21-5p inhibitor or their negative controls (NC). **E** Western blot analysis of the knockdown efficiency of Crim1 siRNAs in VSMCs. **F** Protein levels of Runx2, BMP2 and BMP4 in VSMCs treated with vehicle, siCrim1^#3^ or siRNA-NC under 3.5 mM Pi induction as measured by Western blot. **G** ALP activity in VSMCs after treatment with vehicle, siCrim1^#3^ or siRNA-NC under 3.5 mM Pi induction. Data are the mean ± SD of three independent experiments. *p < 0.05, **p < 0.01

To determine the molecular mechanism through which miR-21-5p promotes the calcification of VSMCs, we used targetscan, pictar, and miRWalk databases to predict the potential target genes of miR-21-5p. 34 genes were identified as miR-21-5p potential target genes (Fig. 5A). A previous study reported that the down expression of Crim1 could enhance the osteogenesis of MSCs [62]. In addition, miR-21-5p was predicted to specifically bind to the predicted target region of the Crim1 mRNA (Fig. 5B). Thus, we speculated Crim1 as the most potential gene involved in the miR-21-5p-mediated pro-calcification effect. The luciferase reporter assay revealed that miR-21-5p mimics suppressed the luciferase activity of the Crim1 3’-UTR reporter gene, this effect was abrogated after the putative binding site of miR-21-5p was mutated (Fig. 5C). Western blot results showed that overexpression of miR-21-5p significantly reduced the expression of Crim1 in VSMCs, while inhibition of miR-21-5p showed the opposite effect (Fig. 5D). These data show that Crim1 is the direct target gene of miR-21-5p.

To further explore the role of Crim1 in the calcification of VSMCs, we constructed 3 small interfering RNA of Crim1 (siCrim1) to silence the Crim1 expression in VSMCs. siCrim1^#3^ exhibited the highest silencing efficiency as shown by the western blot (Fig. 5E), thus we selected siCrim1^#3^ to silence the Crim1 expression in subsequent experiments. It was reported that Crim1 exerts its antagonistic effect by interacting with members of the TGF-β superfamily such as BMP2 and BMP4 [62]. Therefore, in addition to detecting the expression of Runx2 and BMP2 in VSMCs, BMP4 expression was also detected. We found that silencing Crim1 gene expression significantly enhanced the calcification of VSMCs (Fig. 5F-G).

Taken together, our results suggest that inhibition of Crim1 via miR-21-5p mediates the pro-calcification effects of AFs^HPi^-Exos on VSMCs.

### Exosomes in vivo tracking and regulate vascular calcification in 5/6 NTP mouse model

**Fig. 6:**
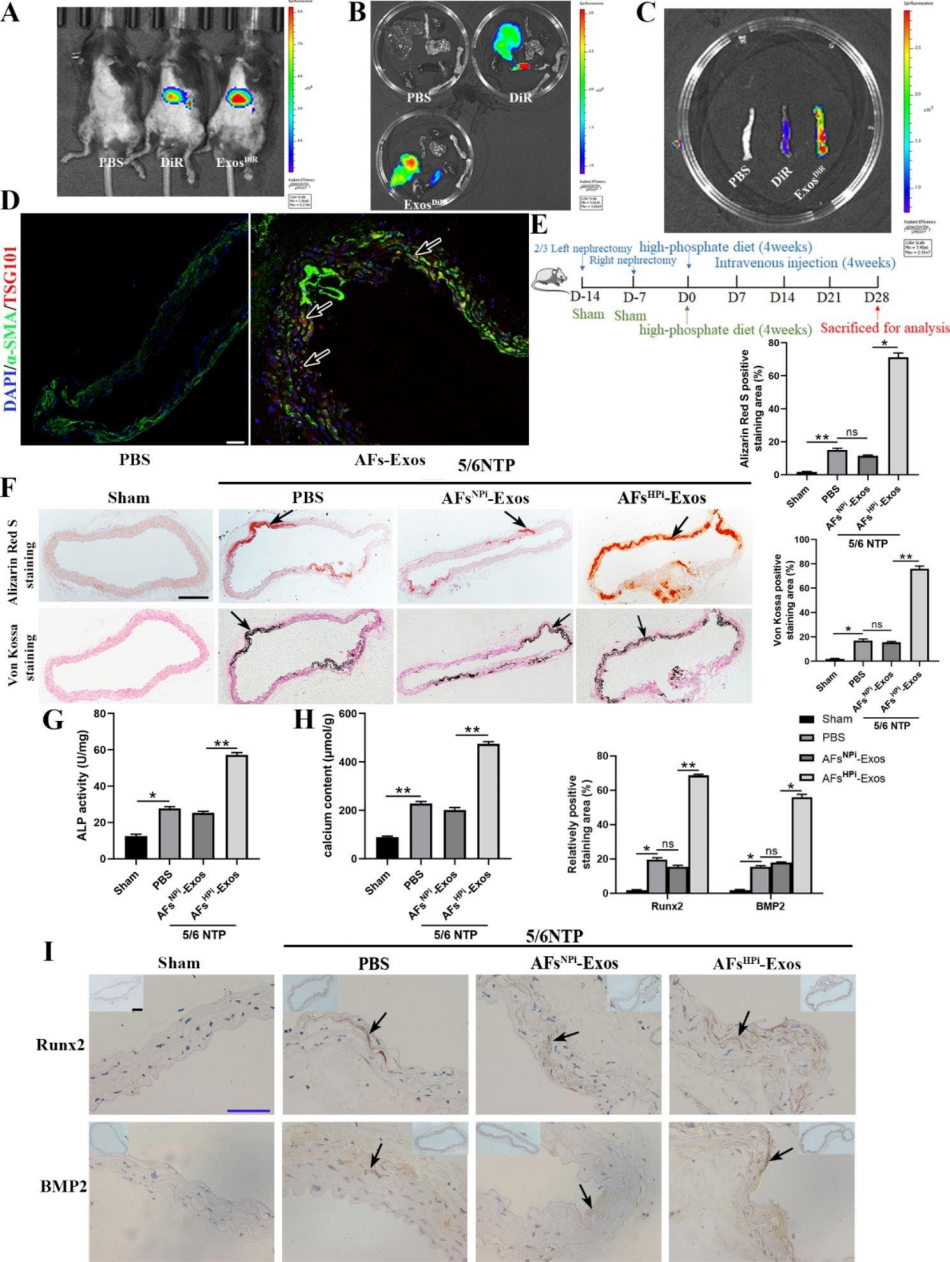
Exosomes in vivo tracking and regulating vascular calcification in 5/6 NTP mouse model. **A** Representative in vivo fluorescent images of 5/6 NTP mice intravenously injected with PBS, DiR or Exos ^DiR^ (100 µg/mice, n = 5 per group) within 12 h. Ex vivo fluorescent imaging of different organs (**B**) and thoracic aortas (**C**) from mice treated with PBS, DiR or Exos ^DiR^ within 12 h. **D** Immunofluorescence analysis of exosome marker TSG101 (red fluorescence) and smooth muscle marker α-SMA (green fluorescence) in thoracic aorta sections from PBS or AFs-Exos treated mice. The arrows indicate TSG101-positive exosomes taken up by the arterial media. The scale bar represents 50 μm. **E** Schematic diagram of the establishment of 5/6 nephrectomy (NTP) mouse model and the intravenous injections of PBS, AFs^NPi^-Exos or AFs^HPi^-Exos (100 µg per mice) every other day for 4 weeks. **F** Alizarin Red S and Von Kossa staining of thoracic aorta sections from sham mice injected with PBS or 5/6 NTP mice (n = 5 per group) injected with PBS, AFs^NPi^-Exos or AFs^HPi^-Exos. The arrows indicate mineralized nodules. The scale bar represents 200 μm. ALP activity (**G**) and calcium content (**H**) of thoracic aortic tissues of mice. **I** Immunohistochemistry analysis of Runx2 and BMP2 expression in the thoracic aorta sections of mice (lower panel) and quantitation of positive staining area (upper panel) are shown. The arrows indicate the positive staining area. Scale bar 200 μm (Black) and 50 μm (Blue). Results are represented by mean ± SD with five replicates for each group. *p < 0.05, **p < 0.01

To investigate whether the exosomes secreted by AFs can be taken up by mice and their distribution in different organs, AFs^HPi^-Exos were labeled with near-infrared dye DiR and intravenously injected into the 5/6 NTP mice. We found that DiR and Exos^DiR^ could be internalized into the mice (Fig. 6A), and the fluorescence signals were mainly distributed in the liver and spleen, while almost no fluorescence signals were detected in other organs, such as the heart, lung, kidney, femur, and thoracic aorta (Fig. 6B). We considered that the fluorescence signal of the liver and spleen might be too strong, masking the weaker fluorescence signal in other organs, thus we imaged the thoracic aorta of the three groups together. Surprisingly, the fluorescence signal appeared in the thoracic aorta of the Exos^DiR^ group, which was stronger than that of the PBS and DiR group (Fig. 6C). We performed double immunofluorescence staining for the exosomal marker TSG101 and the smooth muscle marker α-SMA on thoracic aorta sections from PBS or AFs-Exos treated 5/6 NTP mice. We observed more red fluorescence in the arterial media of the mice in the AFs-Exos group compared with the PBS group (Fig. 6D), indicating that exogenous AFs-Exos could be taken up by the mouse arterial media.

5/6 nephrectomy plus a high-phosphorus diet is a widely used method to develop CRF-induced vascular calcification in mice [2, 39, 63]. One week after nephrectomy, mice in sham and 5/6NTP groups were fed the high-phosphorus diet to accelerate vascular calcification. PBS were intravenously injected into the sham mice, and PBS, AFs^NPi^-Exos or AFs^HPi^-Exos were injected into the 5/6NTP mice every other day. The mice were sacrificed 4 weeks later, and the thoracic aortas were taken for analysis (Fig. 6E). Alizarin Red S and Von Kossa positive staining areas, ALP activity, calcium content as well as Runx2 and BMP2 expression levels in the thoracic aortas of mice were obviously increased in the 5/6 NTP group compared with the sham group (Fig. 6F-I), suggesting that the mouse model of vascular calcification was successfully established. Furthermore, compared with AFs^NPi^-Exos, AFs^HPi^-Exos aggravated 5/6 NTP-induced vascular calcification (Fig. 6F-I). Collectively, our findings reveal that AFs^HPi^-Exos accelerated vascular calcification in vivo.

### MiR-21-5p enriched AFs-Exos promote vascular calcification in 5/6 NTP mouse model

**Fig. 7:**
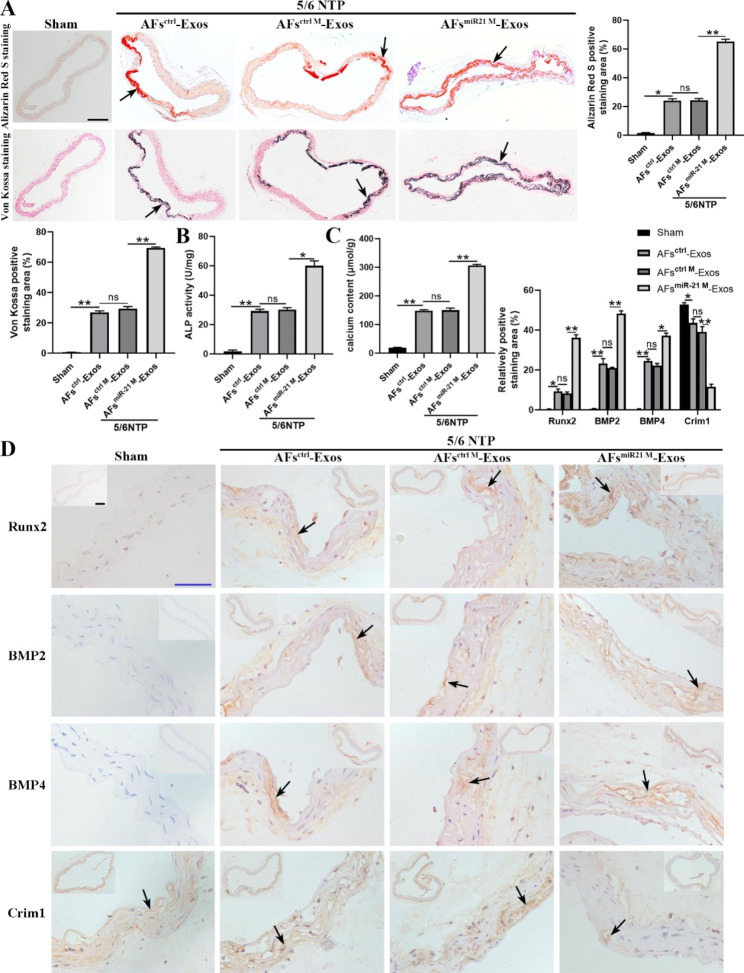
miR-21-5p enriched AFs-Exos promote vascular calcification in 5/6 NTP mouse model. **A** Alizarin Red S and Von Kossa staining images and quantification of the percentage of staining positive areas in thoracic aorta sections from sham mice injected with AFs^ctrl^-Exos or 5/6 NTP mice injected with AFs^ctrl^-Exos, AFs^ctrl M^-Exos or AFs^miR21 M^-Exos (n = 5 per group). The arrows indicate mineralized nodules. The scale bar represents 200 μm. The ALP activity (**B**) and calcium content (**C**) of thoracic aortic tissues normalized to total protein contents. **D** Runx2, BMP2, BMP4 and Crim1 immunohistochemical staining in thoracic aortic tissues and quantitation of positive staining area are shown. The arrows indicate the positive staining area. Scale bar 200 μm (Black) and 50 μm (Blue). Results are represented by mean ± SD with five replicates for each group. *p < 0.05, **p < 0.01

We further explored whether exosomes could regulate vascular calcification by transporting miR-21-5p in vivo. We first transfected AFs with miR-21-5p mimics to overexpress miR-21-5p, and then isolated the exosomes from the AFs-conditioned medium to treat mice. Subsequently, the mice in the sham group were injected with AFs^ctrl^-Exos and the 5/6 NTP-induced mice were injected with AFs^ctrl^-Exos, AFs^ctrl M^-Exos, and AFs^miR21 M^-Exos via tail vein as previously described. We found that 5/6 NTP obviously induced the vascular calcification in mice compared with the sham operation, moreover, AFs^miR21 M^-Exos aggravated the vascular calcification of 5/6 NTP mice, as evidenced by the increases in Alizarin Red S and Von Kossa positive staining areas, ALP activity, calcium content as well as Runx2 and BMP2 expression levels (Fig. 7A-D). Previous in vitro experiments demonstrated that miR-21-5p enhanced the calcification of VSMCs by regulating the BMP2/BMP4/Crim1 pathway (Fig. 5). Consistently, immunohistochemical staining revealed that the aortas of 5/6 NTP mice had higher BMP4 expression and lower Crim1 expression compared to the sham group, and AFs^miR21 M^-Exos significantly increased BMP4 expression and decreased Crim1 expression in the aortas of 5/6 NTP mice (Fig. 7D). Furthermore, BMP4 expression was increased, while the Crim1 expression was significantly decreased in radial artery sections of CRF patients (Additional file 1: Fig. S4). Our results suggest that exosomal miR-21-5p exerts pro-calcification effects by inhibiting Crim1 expression in vivo.

## Discussion

In the present study, we found that exosomes secreted by high phosphorus-induced AFs enriched miR-21-5p, then exosomes were taken up by neighboring VSMCs and transported the miR-21-5p to VSMCs. miR-21-5p promotes the calcification of VSMCs by inhibiting the expression of the downstream target gene Crim1. In addition, a 5/6 NTP plus high-phosphorus diet was used to construct a mouse model of CRF with vascular calcification. In vivo experiments confirmed that AFs-Exos could be taken up by mouse thoracic aortas and vascular calcification in mice with CRF could be obviously aggravated by AFs^HPi^-Exos and AFs^miR21 M^-Exos. Our study highlights the role of the crosstalk between AFs and VSMCs in the development of vascular calcification under the high phosphorus microenvironment. Combined with our previous studies, which showed the communication between ECs and VSMCs, VSMCs with adjacent VSMCs, these experiments construct the theory of vascular wall microenvironment.

In patients with CRF, serum phosphorus is significantly increased due to renal excretion dysfunction. In recent years, many studies have confirmed that high phosphorus is closely related to vascular calcification. Both intimal and medial calcifications occur in patients with CRF, but arterial medial calcification is more common [64]. Studies have shown that hyperphosphatemia can influence the fate of VSMCs by transporting phosphate into VSMCs in a phosphate transporter (Pit1/Pit2)-dependent, independent (in nanoparticle form) or calpain particle (CPP) manner, thereby activating the intracellular pro-calcification signaling pathway and increasing the expression of osteogenic markers Runx2, MSX2, osteogenesis-related transcription factor antibody (osterix), osteopontin and ALP [65, 66]. The main roles of hyperphosphatemia in vascular calcification include: inducing the transition of VSMCs from contractile to osteochondral phenotype and mineralization of the extracellular matrix of VSMCs, inducing apoptosis of VSMCs, inhibiting the differentiation of monocytes or macrophages to osteoclast-like cells, increasing fibroblast growth factor 23 (FGF23) levels, and decreasing Klotho expression [67]. Numerous studies have focused on the direct pro-calcification effect of high phosphate on VSMCs. However, in the complex high-phosphorus microenvironment in vivo, other cells are also affected by high phosphate, so high phosphate may play an indirect role in VSMCs by affecting the function of other cells. The arterial media is composed of VSMCs, while the adventitia is mainly composed of AFs, and there are nutrient vessels in the adventitia of the arteries. Previous studies reported that bioactive molecules secreted by AFs could regulate the proliferation and migration of VSMCs [50, 68]. Therefore, we hypothesized that after receiving the high phosphorus signal in the nutrient vessels, AFs secrete bioactive molecules to affect the calcification of VSMCs. Based on the previous studies [24, 39, 63, 69], we simulated the high phosphorus state in vivo with 3.5 mM inorganic phosphorus and used 0.9 mM inorganic phosphorus as the normal phosphorus control in vitro. Our experiment found that compared with the control group, the conditioned media of HPi-induced AFs significantly enhanced the calcification of VSMCs, but after the synthesis or secretion of exosomes was blocked by GW4869, this pro-calcification effect was partially abolished. Thus, we speculated that the role of AFs^HPi^ conditioned media in promoting VSMCs calcification may be partially mediated by exosomes. Nevertheless, we found that the pretreatment of GW4869 did not completely block the calcification of VSMCs in the high-phosphorus microenvironment, this may be due to the changes in the composition and content of soluble factors secreted by AFs after stimulation by high phosphorus. The secretion of these soluble factors cannot be blocked by GW4869, but may also play a role in the calcification of VSMCs. Other soluble factors that changed after high phosphorus stimulation deserve further exploration in the future.

In recent years, exosomes have become a research hotspot, and their biological functions have received more and more attention. Exosomes are extracellular vesicles with a diameter of 30–150 nm that are involved in intercellular communication [32]. Exosomes can transport miRNAs from parental cells to target cells, and then affect the function of target cells by changing the level of miRNAs [70]. Zhu et al. found that miR-21-3p was enriched in exosomes secreted by nicotine-treated macrophages, and miR-21-3p promoted the migration and proliferation of VSMCs by regulating the expression of its target gene PTEN, thus accelerating the development of atherosclerosis [71]. Another study revealed that AFs and VSMCs can communicate through exosomal miRNAs, the increased expression of miR-135a-5p in exosomes derived from AFs of spontaneously hypertensive rats could inhibit the expression of FNDC5 in VSMCs, thereby promoting the proliferation of VSMCs [44]. These studies indicate that exosomes secreted by AFs can directly affect the functions of VSMCs. Our results showed that exosomes secreted by AFs^HPi^ could be taken up by VSMCs and significantly promoted the calcification of VSMCs. In vivo, we confirmed that exosomes secreted by AFs could be taken up by mouse thoracic aorta, and the vascular calcification in CRF mice was aggravated after the intravenous injection of AFs^HPi^-Exos. As we mentioned previously, in the vascular wall microenvironment, high phosphorus not only directly promotes the calcification of VSMCs, but also indirectly acts on ECs and macrophages, and their secreted exosomes also promote the calcification of VSMCs. These ways work together to amplify the calcification promoting effect of high phosphorus on VSMCs. It is difficult to prove which way is the most critical, our study focused on AFs, which were less studied but indispensable in the high-phosphorus microenvironment. Our finding improved the theory of vascular wall microenvironment and more thoroughly explained the specific mechanisms of calcification of VSMCs in the high-phosphorus microenvironment.

MiRNAs are a class of non-coding RNAs with approximately 22 nucleotides, which are important regulators of post-transcriptional gene expression [72], and participate in the regulation of almost all cellular biological processes [73, 74]. MiRNAs can be used as new biomarkers for the diagnosis, prognosis and treatment of various diseases [75–77]. The 5’ seed region (bases 2–8) of miRNAs can bind to the 3’-UTR region of mRNAs to inhibit the expression of target genes by inducing mRNA degradation or translational repression [78–83]. MiR-21-5p was found to be expressed in rat AFs, with higher expression in myofibroblasts (MFs), furthermore, down-expression of miR-21 enhanced the expression of PDCD4 and inhibited the JNK/c-Jun pathway, thereby decreasing the proliferation and increasing the apoptosis of AFs and MFs, while overexpression of miR-21 had the opposite effects [60]. Macrophages may stimulate the expression of miR-21 in VSMCs and AFs by releasing TNF-α, thereby promoting the proliferation and migration of VSMCs and AFs [57]. These studies suggest that miR-21-5p is expressed in VSMCs and AFs and plays an important role in their biological functions. In addition, studies confirmed that miR-21-5p can positively regulate osteogenic differentiation. Valenti et al. found that miR-21-5p upregulated the level of Runx2 by targeting PTEN and SMAD7 genes, and promoted the osteogenic differentiation of mesenchymal stem cells [52]. Consistently, hucMSC-Exos containing miR-21-5p augmented the osteogenesis of human osteoblasts hFOB1.19 [61], suggesting that miR-21-5p may be a potential regulator of osteogenesis. However, whether miR-21-5p is involved in the differentiation of VSMCs into osteoblast-like cells remains unclear. In the present study, we found that miR-21-5p was enriched in AFs^HPi^-Exos, and the miR-21-5p expression level in AFs was significantly higher than that in VSMCs, so we finally selected miR-21-5p for further investigation. Our results showed that exosomes secreted by AFs overexpressing miR-21-5p significantly promoted the calcification of VSMCs. However, our study also has some shortcomings, in that transfection of miR-21-5p inhibitor in VSMCs significantly inhibited the pro-calcification effect of AFs^HPi^-Exos, but did not completely eliminate this effect. Therefore, it is difficult to rule out other miRNAs or proteins that carried by AFs^HPi^-Exos that may also play a role in the calcification of VSMCs. We further confirmed that exosomes carrying abundant miR-21-5p promoted vascular calcification in 5/6 NTP mice. However, the miR-21-5p that played this role was exogenous, and we have not directly demonstrated in vivo how AFs secreted miR-21-5p-enriched exosomes to regulate the calcification of VSMCs when the vascular wall is in a high-phosphorus microenvironment. Therefore, we could further construct mice model with miR-21-5p AFs-specific knock-out or knock-in, and further detect the expression of miR-21-5p and the calcification level in mouse arterial media.

Crim1 is an plasma membrane binding protein containing cysteine-rich repeat sequences (CRRs) and an insulin-like growth factor binding protein motif [84, 85]. Crim1 gene is most abundantly expressed in the placenta and is expressed in the pancreas, kidney, skeletal muscle, lung, brain, heart and other organs, but not in the liver. The Crim1 gene is also expressed in blood vessels and is involved in the formation and maintenance of capillaries [86]. Fan et al. found that Crim1 was highly expressed in vascular ECs, and conditional knockout of the Crim1 gene in vascular ECs resulted in delayed vasodilation and reduced vascular density [87]. In addition, Nyström et al. detected the expression of Crim1 in primary human pulmonary artery VSMCs, suggesting that Crim1 may play a role in the growth and maintenance of vascular smooth muscle [88]. Crim1 interacts with members of the TGF-β superfamily, such as bone morphogenetic proteins BMP2, BMP4, and BMP7, through its CRR domain, and exerts an antagonistic effect by regulating the transformation of BMP preproteins into mature proteins and the transfer of BMP to the cell surface [85]. It was reported that miR-20a and miR-20b activated the BMPs/Runx2 pathway by inhibiting the expression of PPARγ, Bambi and Crim1 genes, and silencing the expression of Crim1 promoted the osteogenic differentiation of mesenchymal stem cells [62, 89]. However, the targeting relationship between miR-21-5p and the Crim1 gene, and the effect of Crim1 on the calcification of VSMCs have not been elucidated. In our study, we confirmed that miR-21-5p promoted the calcification of VSMCs by targeting Crim1. Moreover, we found that AFs^miR21 M^-Exos reduced the Crim1 expression in the arteries of 5/6 NTP mice. This was consistent with the decreased Crim1 expression in radial arteries of CRF patients. These findings reveal that exosomal miR-21-5p aggravates arterial medial calcification by inhibiting the expression of Crim1.

Studies have shown that exosomes can be used as natural nanocarriers of small molecule compounds due to their excellent biocompatibility and low toxicity, and through genetic engineering modification, exosomes can specifically target a certain tissue to play a more effective therapeutic role [90, 91]. Guo et al. reported that GLG1-modified exosomes carrying Wnt agonist 1 can specifically target BMSCs and promote bone formation [90]. CXCR4-expressing NIH-3T3 cells-derived exosomes have bone-targeting properties, and can mitigate aging-related bone loss by transporting the antagomir-188 into BMSCs [91]. Therefore, in the future, it is expected to develop a genetically engineered strategy to enable antagomir-21-5p-loaded AFs-Exos to specifically target the arterial media, thereby alleviating vascular calcification associated with CRF.

In summary, AFs^HPi^-Exos-mediated transfer of miR-21-5p is probably an important contributor to induce vascular calcification. Strategies targeting AFs^HPi^-Exos could be considered for the treatment of vascular calcification in CRF patients.

## Conclusion

Our findings demonstrate that high phosphate promotes VSMCs calcification by increasing the level of miR-21-5p in AFs-derived exosomes and inhibiting the expression of the target gene Crim1 in VSMCs. This study revealed that the crosstalk between AFs and VSMCs in the vascular high phosphorus microenvironment is an important mechanism of vascular calcification in patients with CRF. Combined with our previous studies on the communication between ECs and VSMCs and the paracrine effect of VSMCs in the regulation of vascular calcification, the theory of “Vascular Wall Microenvironment” has been refined. Nanomaterials loaded with miR-21-5p-inhibitors or Crim1 might be novel therapeutic agents for vascular calcification in CRF patients.

### Electronic supplementary material

Below is the link to the electronic supplementary material.

Additional file 1: Fig. S1. Radial artery calcification in CRF patients. Fig. S2. MiR-21-5p expression levels in VSMCs and AFs. Fig. S3. Transfection efficiency of miR-21-5p mimics/inhibitor. Fig. S4. Immunohistochemical analysis of BMP4 and Crim1 expression in the radial arteries of ND and CRF patients.

## Data Availability

All data generated or analysed during this study are included in this published article [and its supplementary information files].

## Abbreviations

CRF: Chronic renal failure
ECs: Endothelial cells
VSMCs: Vascular smooth muscle cells
HPi: High phosphorus
AFs: Adventitial fibroblasts
AFs^HPi^-Exos: Exosomes secreted by high phosphorus-induced AFs
AFs^miR21M^-Exos: Exosomes secreted by AFs with overexpression of miR-21-5p
Crim1: Cysteine-rich motor neuron 1
Exos: Exosomes
NPi: 0.9 mM inorganic phosphorus
AFs^NPi^-Exos: Exosomes secreted by 0.9 mM inorganic phosphorus induced AFs
ND: Normal donors
GFR: Glomerular filtration rate
FBS: Fetal bovine serum
TEM: Transmission electron microscopy
DLS: Dynamic light scattering
ProtK: Proteinase K
qRT-PCR: Quantitative real-time PCR
5/6 NTP: 5/6 nephrectomy plus a high-phosphorus diet
AFs^HPi^-CM: Conditioned medium of HPi-induced AFs
ALP: Alkaline phosphatase
AFs-Exos: Exosomes from AFs-conditioned medium
BMSCs: Bone marrow mesenchymal stromal cells
siCrim1: Small interfering RNA of Crim1
NC: Negative controls
CPP: Calpain particle
FGF23: Fibroblast growth factor 23
MFs: Myofibroblasts
CRR: Cysteine-rich repeat sequences

## References

[1] Villa-Bellosta R, Egido J (2017). Phosphate, pyrophosphate, and vascular calcification: a question of balance. Eur Heart J.

[2] Xu F, Zhong JY, Lin X, Shan SK, Guo B, Zheng MH, Wang Y, Li F, Cui RR, Wu F (2020). Melatonin alleviates vascular calcification and ageing through exosomal miR-204/miR-211 cluster in a paracrine manner. J Pineal Res.

[3] Kraler S, Blaser MC, Aikawa E, Camici GG, Lüscher TF (2022). Calcific aortic valve disease: from molecular and cellular mechanisms to medical therapy. Eur Heart J.

[4] Zhang H, Li G, Yu X, Yang J, Jiang A, Cheng H, Fu J, Liang X, Liu J, Lou J (2023). Progression of vascular calcification and clinical outcomes in patients receiving maintenance Dialysis. JAMA Netw Open.

[5] Demer LL, Tintut Y (2008). Vascular calcification: pathobiology of a multifaceted disease. Circulation.

[6] Mace ML, Gravesen E, Nordholm A, Egstrand S, Morevati M, Nielsen C, Kjaer A, Behets G, D’Haese P, Olgaard K, Lewin E (2021). Chronic kidney Disease-Induced Vascular Calcification impairs bone metabolism. J Bone Miner Res.

[7] Zheng G, Zhao Y, Li Z, Hua Y, Zhang J, Miao Y, Guo Y, Li L, Shi J, Dong Z (2023). GLSP and GLSP-derived triterpenes attenuate atherosclerosis and aortic calcification by stimulating ABCA1/G1-mediated macrophage cholesterol efflux and inactivating RUNX2-mediated VSMC osteogenesis. Theranostics.

[8] Lin X, Xiang QY, Li S, Song WL, Wang YJ, Ni YQ, Zhao Y, Li C, Wang Y, Li HH (2023). BMF-AS1/BMF promotes Diabetic Vascular calcification and aging both in Vitro and in vivo. Aging Dis.

[9] Thi Nguyen N, Thi Nguyen T, Nguyen HT, Lee JM, Kim MJ, Qi XF, Cha SK, Lee IK, Park KS (2023). Inhibition of mitochondrial phosphate carrier prevents high phosphate-induced superoxide generation and vascular calcification. Exp Mol Med.

[10] Lin X, Li S, Wang YJ, Wang Y, Zhong JY, He JY, Cui XJ, Zhan JK, Liu YS. Exosomal Notch3 from high glucose-stimulated endothelial cells regulates vascular smooth muscle cells calcification/aging. Life Sci. 2019;232116582. 10.1016/j.lfs.2019.116582. 10.1016/j.lfs.2019.116582 31220525

[11] Zhao MM, Xu MJ, Cai Y, Zhao G, Guan Y, Kong W, Tang C, Wang X (2011). Mitochondrial reactive oxygen species promote p65 nuclear translocation mediating high-phosphate-induced vascular calcification in vitro and in vivo. Kidney Int.

[12] Drüeke TB (2008). Arterial intima and media calcification: distinct entities with different pathogenesis or all the same?. Clin J Am Soc Nephrol.

[13] Durham AL, Speer MY, Scatena M, Giachelli CM, Shanahan CM (2018). Role of smooth muscle cells in vascular calcification: implications in atherosclerosis and arterial stiffness. Cardiovasc Res.

[14] Koide T, Mandai S, Kitaoka R, Matsuki H, Chiga M, Yamamoto K, Yoshioka K, Yagi Y, Suzuki S, Fujiki T (2023). Circulating Extracellular vesicle-propagated microRNA signature as a vascular calcification factor in chronic kidney disease. Circ Res.

[15] Carney EF (2020). The impact of chronic kidney disease on global health. Nat Rev Nephrol.

[16] Moe SM, Chen NX (2004). Pathophysiology of vascular calcification in chronic kidney disease. Circ Res.

[17] Bundy JD, Chen J, Yang W, Budoff M, Go AS, Grunwald JE, Kallem RR, Post WS, Reilly MP, Ricardo AC, et al. Risk factors for progression of coronary artery calcification in patients with chronic kidney disease: the CRIC study. Atherosclerosis. 2018;27153–60. 10.1016/j.atherosclerosis.2018.02.009. 10.1016/j.atherosclerosis.2018.02.009 PMC5864458 29459266

[18] Zanoli L, Lentini P, Briet M, Castellino P, House AA, London GM, Malatino L, McCullough PA, Mikhailidis DP, Boutouyrie P (2019). Arterial stiffness in the Heart Disease of CKD. J Am Soc Nephrol.

[19] Kanbay M, Goldsmith D, Akcay A, Covic A (2009). Phosphate - the silent stealthy cardiorenal culprit in all stages of chronic kidney disease: a systematic review. Blood Purif.

[20] Shanahan CM, Crouthamel MH, Kapustin A, Giachelli CM (2011). Arterial calcification in chronic kidney disease: key roles for calcium and phosphate. Circ Res.

[21] Nagy A, Pethő D, Gesztelyi R, Juhász B, Balla G, Szilvássy Z, Balla J, Gáll T. BGP-15 inhibits hyperglycemia-aggravated VSMC Calcification Induced by high phosphate. Int J Mol Sci. 2021;22(17). 10.3390/ijms22179263. 10.3390/ijms22179263 PMC8431374 34502172

[22] Nguyen NT, Nguyen TT, Da Ly D, Xia JB, Qi XF, Lee IK, Cha SK, Park KS (2020). Oxidative stress by ca(2+) overload is critical for phosphate-induced vascular calcification. Am J Physiol Heart Circ Physiol.

[23] Cao YC, Shan SK, Guo B, Li CC, Li FX, Zheng MH, Xu QS, Wang Y, Lei LM, Tang KX, et al. Histone lysine methylation modification and its role in vascular calcification. Front Endocrinol (Lausanne). 2022;13863708. 10.3389/fendo.2022.863708. 10.3389/fendo.2022.863708 PMC9243330 35784574

[24] Lin X, Shan SK, Xu F, Zhong JY, Wu F, Duan JY, Guo B, Li FX, Wang Y, Zheng MH (2022). The crosstalk between endothelial cells and vascular smooth muscle cells aggravates high phosphorus-induced arterial calcification. Cell Death Dis.

[25] Li Q, Zhang C, Shi J, Yang Y, Xing X, Wang Y, Zhan X, Wang L, Xu G, He F. High-phosphate-stimulated macrophage-derived Exosomes promote vascular calcification via let-7b-5p/TGFBR1 Axis in chronic kidney disease. Cells. 2022;12(1). 10.3390/cells12010161. 10.3390/cells12010161 PMC9818696 36611957

[26] Wu YY, Shan SK, Lin X, Xu F, Zhong JY, Wu F, Duan JY, Guo B, Li FX, Wang Y, et al. Cellular Crosstalk in the Vascular Wall Microenvironment: the role of Exosomes in vascular calcification. Front Cardiovasc Med. 2022;9912358. 10.3389/fcvm.2022.912358. 10.3389/fcvm.2022.912358 PMC9168031 35677687

[27] Guo B, Shan SK, Xu F, Lin X, Li FX, Wang Y, Xu QS, Zheng MH, Lei LM, Li CC (2022). Protective role of small extracellular vesicles derived from HUVECs treated with AGEs in diabetic vascular calcification. J Nanobiotechnol.

[28] Crescitelli R, Lässer C, Szabó TG, Kittel A, Eldh M, Dianzani I, Buzás EI, Lötvall J. Distinct RNA profiles in subpopulations of extracellular vesicles: apoptotic bodies, microvesicles and exosomes. J Extracell Vesicles. 2013;2. 10.3402/jev.v2i0.20677. 10.3402/jev.v2i0.20677 PMC3823106 24223256

[29] Han QF, Li WJ, Hu KS, Gao J, Zhai WL, Yang JH, Zhang SJ (2022). Exosome biogenesis: machinery, regulation, and therapeutic implications in cancer. Mol Cancer.

[30] Février B, Raposo G (2004). Exosomes: endosomal-derived vesicles shipping extracellular messages. Curr Opin Cell Biol.

[31] Wang ZX, Luo ZW, Li FX, Cao J, Rao SS, Liu YW, Wang YY, Zhu GQ, Gong JS, Zou JT (2022). Aged bone matrix-derived extracellular vesicles as a messenger for calcification paradox. Nat Commun.

[32] van Niel G, D’Angelo G, Raposo G (2018). Shedding light on the cell biology of extracellular vesicles. Nat Rev Mol Cell Biol.

[33] Isaac R, Reis FCG, Ying W, Olefsky JM (2021). Exosomes as mediators of intercellular crosstalk in metabolism. Cell Metab.

[34] Keller S, Ridinger J, Rupp AK, Janssen JW, Altevogt P. Body fluid derived exosomes as a novel template for clinical diagnostics. J Transl Med. 2011;986. 10.1186/1479-5876-9-86. 10.1186/1479-5876-9-86 PMC3118335 21651777

[35] Xue M, Chen W, Xiang A, Wang R, Chen H, Pan J, Pang H, An H, Wang X, Hou H, Li X (2017). Hypoxic exosomes facilitate bladder tumor growth and development through transferring long non-coding RNA-UCA1. Mol Cancer.

[36] Lin X, Zhu T, Xu F, Zhong JY, Li F, Shan SK, Wu F, Guo B, Zheng MH, Wang Y, et al. Plasma exosomes derived from patients with end-stage renal disease and renal transplant recipients have different Effects on vascular calcification. Front Cell Dev Biol. 2020;8618228. 10.3389/fcell.2020.618228. 10.3389/fcell.2020.618228 PMC7876285 33585452

[37] Xu F, Zhong JY, Guo B, Lin X, Wu F, Li FX, Shan SK, Zheng MH, Wang Y, Xu QS, et al. H19 promotes osteoblastic transition by acting as ceRNA of mir-140-5p in vascular smooth muscle cells. Front Cell Dev Biol. 2022;10774363. 10.3389/fcell.2022.774363. 10.3389/fcell.2022.774363 PMC8859097 35198556

[38] Wu F, Li F, Lin X, Xu F, Cui RR, Zhong JY, Zhu T, Shan SK, Liao XB, Yuan LQ, Mo ZH (2019). Exosomes increased angiogenesis in papillary thyroid cancer microenvironment. Endocr Relat Cancer.

[39] Lin X, Xu F, Cui RR, Xiong D, Zhong JY, Zhu T, Li F, Wu F, Xie XB, Mao MZ (2018). Arterial calcification is regulated Via an miR-204/DNMT3a Regulatory Circuit both in Vitro and in female mice. Endocrinology.

[40] Xu F, Li FX, Lin X, Zhong JY, Wu F, Shan SK, Tan CM, Yuan LQ, Liao XB (2019). Adipose tissue-derived omentin-1 attenuates arterial calcification via AMPK/Akt signaling pathway. Aging.

[41] Opdebeeck B, Neven E, Millán JL, Pinkerton AB, D’Haese PC, Verhulst A. Chronic kidney Disease-Induced arterial media calcification in rats prevented by tissue non-specific alkaline phosphatase substrate supplementation rather than inhibition of the enzyme. Pharmaceutics. 2021;13(8). 10.3390/pharmaceutics13081138. 10.3390/pharmaceutics13081138 PMC8399849 34452102

[42] Neven E, D’Haese PC (2011). Vascular calcification in chronic renal failure: what have we learned from animal studies?. Circ Res.

[43] Leskinen Y, Salenius JP, Lehtimäki T, Huhtala H, Saha H (2002). The prevalence of peripheral arterial disease and medial arterial calcification in patients with chronic renal failure: requirements for diagnostics. Am J Kidney Dis.

[44] Tong Y, Ye C, Zheng F, Bo JH, Wu LL, Han Y, Zhou YB, Xiong XQ, Chen Q, Li YH, et al. Extracellular vesicle-mediated miR135a-5p transfer in hypertensive rat contributes to vascular smooth muscle cell proliferation via targeting FNDC5. Vascul Pharmacol. 2021;140106864. 10.1016/j.vph.2021.106864. 10.1016/j.vph.2021.106864 33865997

[45] Liu C, Liu AS, Zhong D, Wang CG, Yu M, Zhang HW, Xiao H, Liu JH, Zhang J, Yin K, Circular (2021). RNA AFF4 modulates osteogenic differentiation in BM-MSCs by activating SMAD1/5 pathway through miR-135a-5p/FNDC5/Irisin axis. Cell Death Dis.

[46] Hrdlicka HC, Pereira RC, Shin B, Yee SP, Deymier AC, Lee SK, Delany AM. Inhibition of mir-29-3p isoforms via tough decoy suppresses osteoblast function in homeostasis but promotes intermittent parathyroid hormone-induced bone anabolism. Bone. 2021;143115779. 10.1016/j.bone.2020.115779. 10.1016/j.bone.2020.115779 PMC7770763 33253931

[47] Cao Y, Lv Q, Li Y, Astragaloside IV (2021). Improves tibial defect in rats and promotes proliferation and osteogenic differentiation of hBMSCs through MiR-124-3p.1/STAT3 Axis. J Nat Prod.

[48] Xu X, Chen Y, Tan B, Wang D, Yuan Z, Wang F (2020). Circular RNA circ_0011269 sponges miR-122 to regulate RUNX2 expression and promotes osteoporosis progression. J Cell Biochem.

[49] Song JJ, Yang M, Liu Y, Song JW, Wang J, Chi HJ, Liu XY, Zuo K, Yang XC, Zhong JC. MicroRNA-122 aggravates angiotensin II-mediated apoptosis and autophagy imbalance in rat aortic adventitial fibroblasts via the modulation of SIRT6-elabela-ACE2 signaling. Eur J Pharmacol. 2020;883173374. 10.1016/j.ejphar.2020.173374. 10.1016/j.ejphar.2020.173374 PMC7364171 32682786

[50] Ren XS, Tong Y, Qiu Y, Ye C, Wu N, Xiong XQ, Wang JJ, Han Y, Zhou YB, Zhang F (2020). MiR155-5p in adventitial fibroblasts-derived extracellular vesicles inhibits vascular smooth muscle cell proliferation via suppressing angiotensin-converting enzyme expression. J Extracell Vesicles.

[51] He X, Wang Z, Wei L, Cheng X, Chen L, Gao F, Jiang H (2020). Indoxyl sulfate promotes osteogenic differentiation of vascular smooth muscle cells by mir-155-5p-dependent downregulation of matrix gla protein via ROS/NF-κB signaling. Exp Cell Res.

[52] Valenti MT, Deiana M, Cheri S, Dotta M, Zamboni F, Gabbiani D, Schena F, Dalle Carbonare L, Mottes M. Physical Exercise modulates miR-21-5p, miR-129-5p, miR-378-5p, and mir-188-5p expression in Progenitor cells promoting Osteogenesis. Cells. 2019;8(7). 10.3390/cells8070742. 10.3390/cells8070742 PMC6678390 31330975

[53] Kozlova A, Pachera E, Maurer B, Jüngel A, Distler JHW, Kania G, Distler O (2019). Regulation of Fibroblast apoptosis and proliferation by MicroRNA-125b in systemic sclerosis. Arthritis Rheumatol.

[54] John AA, Prakash R, Singh D (2019). miR-487b-3p impairs osteoblastogenesis by targeting notch-regulated ankyrin-repeat protein (Nrarp). J Endocrinol.

[55] Chao CT, Yeh HY, Yuan TH, Chiang CK, Chen HW (2019). MicroRNA-125b in vascular diseases: an updated systematic review of pathogenetic implications and clinical applications. J Cell Mol Med.

[56] Zhang H, Wang D, Li M, Plecitá-Hlavatá L, D’Alessandro A, Tauber J, Riddle S, Kumar S, Flockton A, McKeon BA (2017). Metabolic and proliferative state of vascular adventitial fibroblasts in pulmonary hypertension is regulated through a MicroRNA-124/PTBP1 (polypyrimidine tract binding protein 1)/Pyruvate kinase muscle Axis. Circulation.

[57] Guo X, Sun M, Dai C, Zhang X, Yin Q, Ling J, Li X, Wu X, Jiang F, Wang J (2017). Macrophage-stimulated microRNA expression in mural cells promotes transplantation-induced neointima formation. Oncotarget.

[58] Luo Y, Dong HY, Zhang B, Feng Z, Liu Y, Gao YQ, Dong MQ, Li ZC (2015). miR-29a-3p attenuates hypoxic pulmonary hypertension by inhibiting pulmonary adventitial fibroblast activation. Hypertension.

[59] Nossent AY, Eskildsen TV, Andersen LB, Bie P, Brønnum H, Schneider M, Andersen DC, Welten SM, Jeppesen PL, Hamming JF (2013). The 14q32 microRNA-487b targets the antiapoptotic insulin receptor substrate 1 in hypertension-induced remodeling of the aorta. Ann Surg.

[60] Wang F, Zhao XQ, Liu JN, Wang ZH, Wang XL, Hou XY, Liu R, Gao F, Zhang MX, Zhang Y, Bu PL (2012). Antagonist of microRNA-21 improves balloon injury-induced rat iliac artery remodeling by regulating proliferation and apoptosis of adventitial fibroblasts and myofibroblasts. J Cell Biochem.

[61] Fang S, Liu Z, Wu S, Chen X, You M, Li Y, Yang F, Zhang S, Lai Y, Liu P (2022). Pro-angiognetic and pro-osteogenic effects of human umbilical cord mesenchymal stem cell-derived exosomal mir-21-5p in osteonecrosis of the femoral head. Cell Death Discov.

[62] Zhang JF, Fu WM, He ML, Xie WD, Lv Q, Wan G, Li G, Wang H, Lu G, Hu X (2011). MiRNA-20a promotes osteogenic differentiation of human mesenchymal stem cells by co-regulating BMP signaling. RNA Biol.

[63] Lin X, Li F, Xu F, Cui RR, Xiong D, Zhong JY, Zhu T, Shan SK, Wu F, Xie XB et al. Aberration methylation of miR-34b was involved in regulating vascular calcification by targeting Notch1. Aging (Albany NY). 2019; 11(10):3182–97. 10.18632/aging.101973. 10.18632/aging.101973 PMC6555467 31129659

[64] Vervloet M, Cozzolino M (2017). Vascular calcification in chronic kidney disease: different bricks in the wall?. Kidney Int.

[65] Chen NX, Moe SM (2012). Vascular calcification: pathophysiology and risk factors. Curr Hypertens Rep.

[66] Lee SJ, Lee IK, Jeon JH. Vascular calcification-new Insights into its mechanism. Int J Mol Sci. 2020;21(8). 10.3390/ijms21082685. 10.3390/ijms21082685 PMC7216228 32294899

[67] Cozzolino M, Ciceri P, Galassi A, Mangano M, Carugo S, Capelli I, Cianciolo G. The key role of phosphate on vascular calcification. Toxins (Basel). 2019;11(4). 10.3390/toxins11040213. 10.3390/toxins11040213 PMC6521180 30970562

[68] Chen Y, Chen Y, Jiang X, Shi M, Yang Z, Chen Z, Hua X, Chen J, Wang Y. Vascular adventitial fibroblasts-derived FGF10 promotes vascular smooth muscle cells Proliferation and Migration in vitro and the Neointima formation in vivo. J Inflamm Res. 2021;142207–23. 10.2147/jir.S305204. 10.2147/JIR.S305204 PMC8164702 34079328

[69] Masumoto A, Sonou T, Ohya M, Yashiro M, Nakashima Y, Okuda K, Iwashita Y, Mima T, Negi S, Shigematsu T (2017). Calcium overload accelerates Phosphate-Induced Vascular Calcification Via Pit-1, but not the calcium-sensing receptor. J Atheroscler Thromb.

[70] Yu X, Odenthal M, Fries JW. Exosomes as miRNA carriers: formation-function-future. Int J Mol Sci. 2016;17(12). 10.3390/ijms17122028. 10.3390/ijms17122028 PMC5187828 27918449

[71] Zhu J, Liu B, Wang Z, Wang D, Ni H, Zhang L, Wang Y (2019). Exosomes from nicotine-stimulated macrophages accelerate atherosclerosis through miR-21-3p/PTEN-mediated VSMC migration and proliferation. Theranostics.

[72] Ghafouri-Fard S, Shoorei H, Mohaqiq M, Majidpoor J, Moosavi MA, Taheri M (2022). Exploring the role of non-coding RNAs in autophagy. Autophagy.

[73] Ferragut Cardoso AP, Banerjee M, Nail AN, Lykoudi A, States JC. miRNA dysregulation is an emerging modulator of genomic instability. Semin Cancer Biol. 2021;76120–31. 10.1016/j.semcancer.2021.05.004. 10.1016/j.semcancer.2021.05.004 PMC8576067 33979676

[74] Fabian MR, Sonenberg N, Filipowicz W. Regulation of mRNA translation and stability by microRNAs. Annu Rev Biochem. 2010;79351–79. 10.1146/annurev-biochem-060308-103103. 10.1146/annurev-biochem-060308-103103 20533884

[75] Sorop A, Constantinescu D, Cojocaru F, Dinischiotu A, Cucu D, Dima SO. Exosomal microRNAs as biomarkers and therapeutic targets for Hepatocellular Carcinoma. Int J Mol Sci. 2021;22(9). 10.3390/ijms22094997. 10.3390/ijms22094997 PMC8125948 34066780

[76] So JBY, Kapoor R, Zhu F, Koh C, Zhou L, Zou R, Tang YC, Goo PCK, Rha SY, Chung HC (2021). Development and validation of a serum microRNA biomarker panel for detecting gastric cancer in a high-risk population. Gut.

[77] Preethi KA, Selvakumar SC, Ross K, Jayaraman S, Tusubira D, Sekar D (2022). Liquid biopsy: exosomal microRNAs as novel diagnostic and prognostic biomarkers in cancer. Mol Cancer.

[78] Weidner J, Bartel S, Kılıç A, Zissler UM, Renz H, Schwarze J, Schmidt-Weber CB, Maes T, Rebane A, Krauss-Etschmann S, Rådinger M (2021). Spotlight on microRNAs in allergy and asthma. Allergy.

[79] Kilikevicius A, Meister G, Corey DR (2022). Reexamining assumptions about miRNA-guided gene silencing. Nucleic Acids Res.

[80] Bartel DP (2004). MicroRNAs: genomics, biogenesis, mechanism, and function. Cell.

[81] Bartel DP (2009). MicroRNAs: target recognition and regulatory functions. Cell.

[82] Filipowicz W, Bhattacharyya SN, Sonenberg N (2008). Mechanisms of post-transcriptional regulation by microRNAs: are the answers in sight?. Nat Rev Genet.

[83] Wu YL, Lin ZJ, Li CC, Lin X, Shan SK, Guo B, Zheng MH, Li F, Yuan LQ, Li ZH (2023). Epigenetic regulation in metabolic diseases: mechanisms and advances in clinical study. Signal Transduct Target Ther.

[84] Sahni V, Itoh Y, Shnider SJ, Macklis JD (2021). Crim1 and Kelch-like 14 exert complementary dual-directional developmental control over segmentally specific corticospinal axon projection targeting. Cell Rep.

[85] Wilkinson L, Kolle G, Wen D, Piper M, Scott J, Little M (2003). CRIM1 regulates the rate of processing and delivery of bone morphogenetic proteins to the cell surface. J Biol Chem.

[86] Glienke J, Sturz A, Menrad A, Thierauch KH (2002). CRIM1 is involved in endothelial cell capillary formation in vitro and is expressed in blood vessels in vivo. Mech Dev.

[87] Fan J, Ponferrada VG, Sato T, Vemaraju S, Fruttiger M, Gerhardt H, Ferrara N, Lang RA (2014). Crim1 maintains retinal vascular stability during development by regulating endothelial cell Vegfa autocrine signaling. Development.

[88] Nyström J, Hultenby K, Ek S, Sjölund J, Axelson H, Jirström K, Saleem MA, Nilsson K, Johansson ME (2009). CRIM1 is localized to the podocyte filtration slit diaphragm of the adult human kidney. Nephrol Dial Transplant.

[89] He J, Zhang JF, Yi C, Lv Q, Xie WD, Li JN, Wan G, Cui K, Kung HF, Yang J (2010). miRNA-mediated functional changes through co-regulating function related genes. PLoS ONE.

[90] Guo J, Wang F, Hu Y, Luo Y, Wei Y, Xu K, Zhang H, Liu H, Bo L, Lv S (2023). Exosome-based bone-targeting drug delivery alleviates impaired osteoblastic bone formation and bone loss in inflammatory bowel diseases. Cell Rep Med.

[91] Hu Y, Li X, Zhang Q, Gu Z, Luo Y, Guo J, Wang X, Jing Y, Chen X, Su J (2021). Exosome-guided bone targeted delivery of Antagomir-188 as an anabolic therapy for bone loss. Bioact Mater.

