# Supplementary material for: Vascular wall microenvironment: exosomes secreted by adventitial fibroblasts induced vascular calcification
Source: J Nanobiotechnology. 2023 Sep 4;21:315. doi: 10.1186/s12951-023-02000-3 (PMC10478424; doi:10.1186/s12951-023-02000-3)
Supplement: Supplementary file 1 — Additional file 1: Fig. S1. Radial artery calcification in CRF patients. Fig. S2. MiR-21-5p expression levels in VSMCs and AFs. Fig. S3. Transfection efficiency of miR-21-5p mimics/inhibitor. Fig. S4. Immunohistochemical analysis of BMP4 and Crim1 expression in the radial arteries of ND and CRF patients. [file 12951_2023_2000_MOESM1_ESM.docx]

**Supplementary Materials**

**Vascular Wall Microenvironment: Exosomes Secreted by Adventitial Fibroblasts Induced Vascular Calcification**

Minghui Zheng^1^, Sukang Shan^1^, Xiao Lin^2^, Feng Xu^1^, Feng Wu^3^, Bei Guo^1^, Fuxingzi Li^1^, Zhiang Zhou^4^, Yi Wang^1^, Limin Lei^1^, Kexin Tang^1^, Jiayue Duan^1^, Yunyun Wu^1^, Yechi Cao^1^, Xiaobo Liao^4^^*^, Lingqing Yuan^1*^


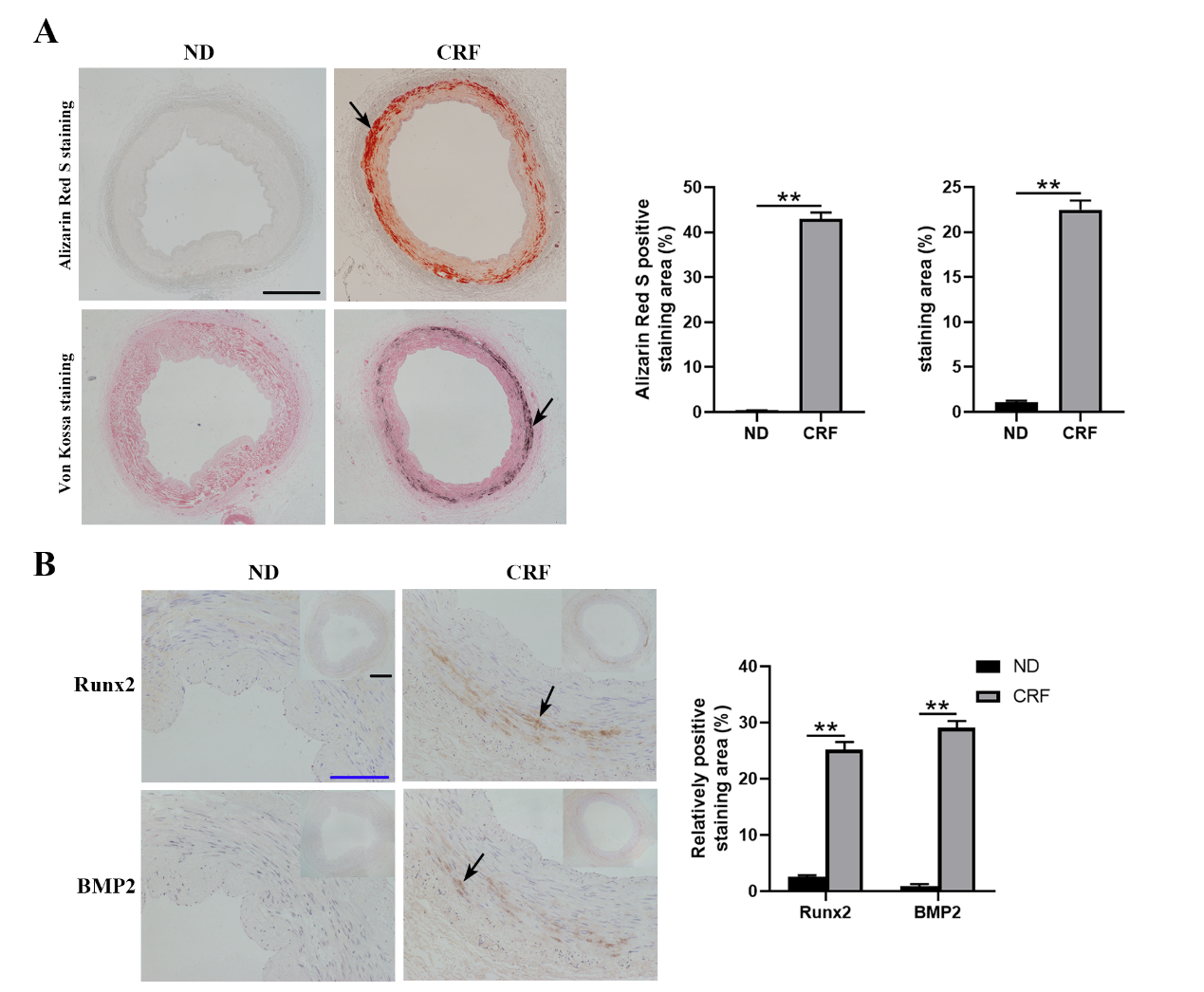


**Fig. S1** Radial artery calcification in CRF patients. **A** Alizarin Red S and Von Kossa staining images and quantification of the percentage of staining positive areas in radial arteries of normal donors (ND) and chronic renal failure (CRF) patients (n = 5 per group). The arrows indicate mineralized nodules. Scale bar represents 500 μm. **B** Immunohistochemical analysis of Runx2 and BMP2 expression in the radial arteries sections of ND and CRF patients. The arrows indicate the positive staining area. Scale bar 500 μm (Black) and 200 μm (Blue). Results are represented by mean ± SD with five replicates for each group. **p* < 0.05, ***p* < 0.01.


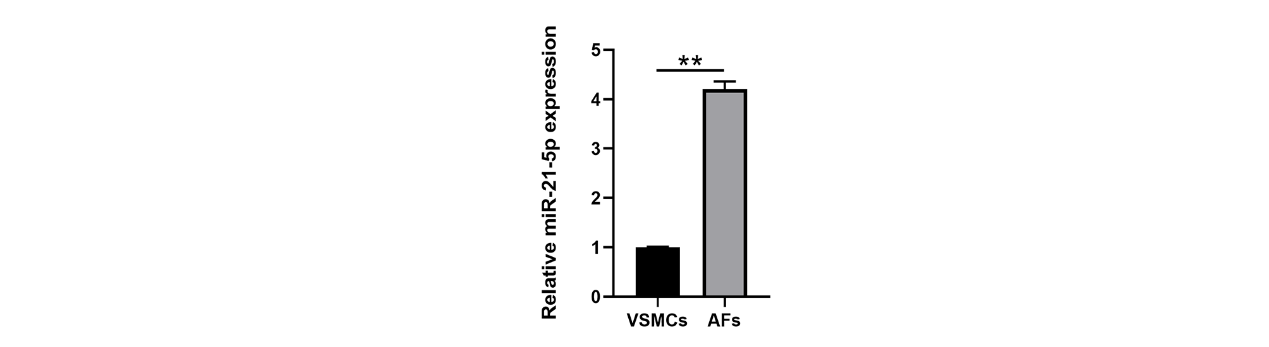


**Fig. S2** MiR-21-5p expression in VSMCs and AFs. qRT-PCR analysis of miR-21-5p expression in VSMCs and AFs. Data are the mean ± SD of three independent experiments. **p* < 0.05, ***p* < 0.01.


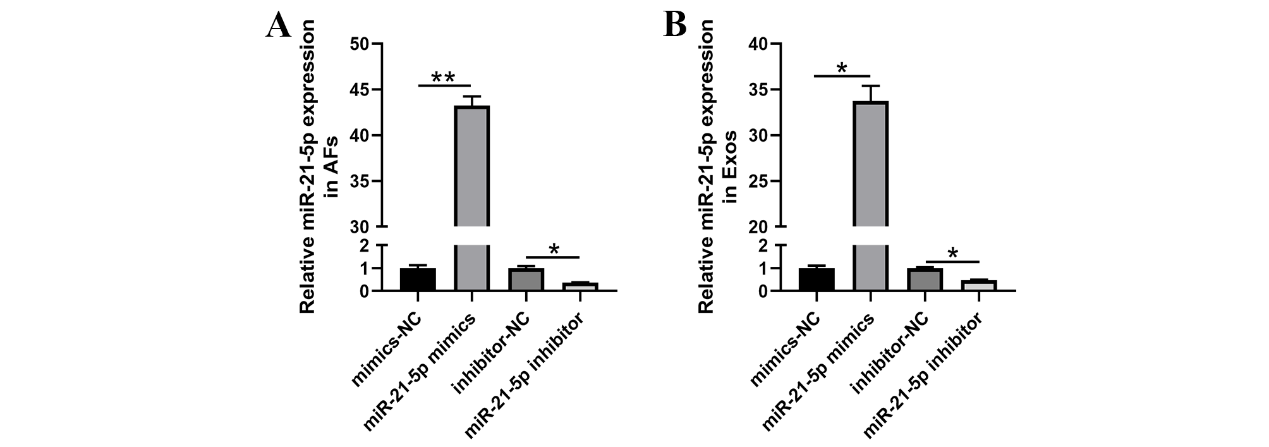


**Fig. S3** Transfection efficiency of miR-21-5p mimics/inhibitor. qRT-PCR analysis of miR-21-5p levels in AFs (**A**) and secreted exosomes (**B**) after AFs were transfected with miR-21-5p mimics, miR-21-5p inhibitor or their negative controls (NC). Data are the mean ± SD of three independent experiments. **p* < 0.05, ***p* < 0.01.


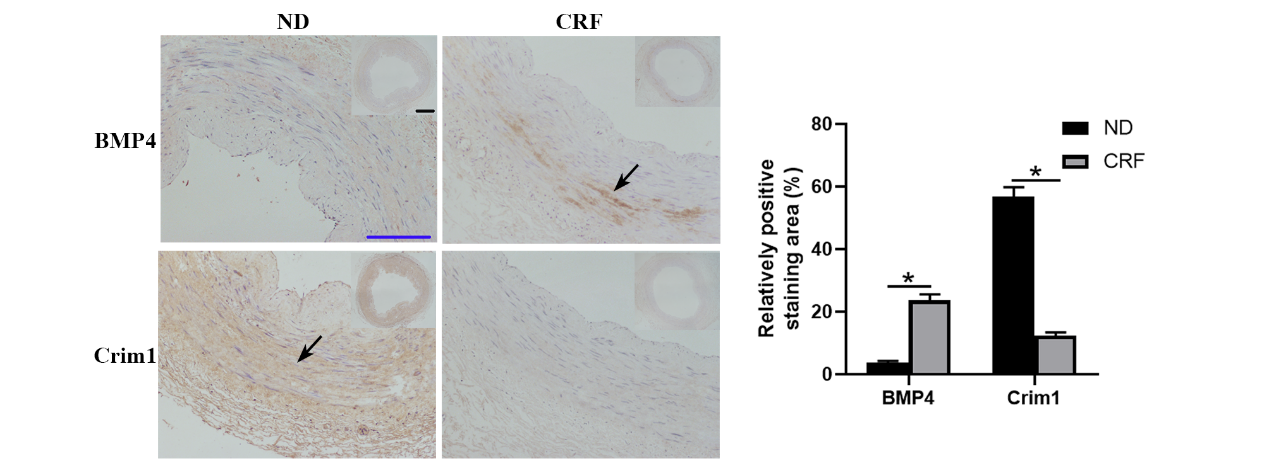


**Fig. S4** Immunohistochemical analysis of BMP4 and Crim1 expression in the radial arteries of ND/CRF patients. The arrows indicate the positive staining area. Scale bar 500 μm (Black) and 200 μm (Blue). Results are represented by mean ± SD with five replicates for each group. **p* < 0.05, ***p* < 0.01.
